# The global distribution of known and undiscovered ant biodiversity

**DOI:** 10.1126/sciadv.abp9908

**Published:** 2022-08-03

**Authors:** Jamie M. Kass, Benoit Guénard, Kenneth L. Dudley, Clinton N. Jenkins, Fumika Azuma, Brian L. Fisher, Catherine L. Parr, Heloise Gibb, John T. Longino, Philip S. Ward, Anne Chao, David Lubertazzi, Michael Weiser, Walter Jetz, Robert Guralnick, Rumsaïs Blatrix, James Des Lauriers, David A. Donoso, Christos Georgiadis, Kiko Gomez, Peter G. Hawkes, Robert A. Johnson, John E. Lattke, Joe A. MacGown, William Mackay, Simon Robson, Nathan J. Sanders, Robert R. Dunn, Evan P. Economo

**Affiliations:** ^1^ Biodiversity and Biocomplexity Unit, Okinawa Institute of Science and Technology, Onna, Okinawa 904-0495, Japan.; ^2^ School of Biological Sciences, The University of Hong Kong, Pokfulam, Hong Kong.; ^3^ Department of Earth and Environment and Kimberly Green Latin American and Caribbean Center, Florida International University, 11200 SW 8th Street, Miami, FL 33199, USA.; ^4^ Entomology, California Academy of Sciences, San Francisco, CA 94118, USA.; ^5^ School of Environmental Sciences, University of Liverpool, Liverpool L69 3GP, UK.; ^6^ Department of Zoology and Entomology, University of Pretoria, Pretoria 0028, South Africa.; ^7^ School of Animal, Plant, and Environmental Sciences, University of the Witwatersrand, Johannesburg, Wits 2050, South Africa; ^8^ Department of Ecology, Environment and Evolution, and Center for Future Landscapes, La Trobe University, Bundoora, Victoria 3086, Australia.; ^9^ School of Biology, University of Utah, Salt Lake City, UT 84112, USA.; ^10^ Department of Entomology and Nematology, University of California, Davis, Davis, CA 95616, USA.; ^11^ Institute of Statistics, National Tsing Hua University, Hsin-Chu 30043, Taiwan.; ^12^ Museum of Comparative Zoology, Harvard University, 26 Oxford Street, Cambridge, MA 02138, USA.; ^13^ Department of Biology and Geographical Ecology Group, University of Oklahoma, Norman, OK 73019, USA.; ^14^ Center for Biodiversity and Global Change and Department of Ecology and Evolutionary Biology, Yale University, New Haven, CT 06511, USA.; ^15^ Florida Museum of Natural History, University of Florida, Gainesville, FL 32611, USA.; ^16^ CEFE, Univ Montpellier, CNRS, EPHE, IRD, Montpellier, France.; ^17^ Department of Biology, Chaffey College, Rancho Cucamonga, CA 91737, USA.; ^18^ Departamento de Biología, Escuela Politécnica Nacional, Quito, Ecuador.; ^19^ Section of Zoology–Marine Biology, Department of Biology, National and Kapodistrian University of Athens, Zografou 15772, Greece.; ^20^ Castelldefels, Barcelona, Spain.; ^21^ AfriBugs CC, 341 27th Avenue, Villieria, Pretoria, Gauteng Province 0186, South Africa.; ^22^ Department of Biological Sciences, University of Venda, Thohoyandou, Limpopo Province, South Africa.; ^23^ School of Life Sciences, Arizona State University, Tempe, AZ 852787-4501, USA.; ^24^ Department of Zoology, Universidade Federal do Paraná, Curitiba, CEP 81531-980, PR, Brazil.; ^25^ Department of Molecular Biology, Biochemistry, Entomology, and Plant Pathology, Mississippi State University, Mississippi State, MS 39762, USA.; ^26^ Biodiversity Collections, Department of Biological Sciences, University of Texas, El Paso, TX, 79968, USA.; ^27^ College of Science and Engineering, Central Queensland University, Townsville, QLD 4812, Australia.; ^28^ Department of Ecology and Evolutionary Biology, University of Michigan, Ann Arbor, MI, USA.; ^29^ Department of Applied Ecology, North Carolina State University, Raleigh, NC 27607, USA.; ^30^ Radcliffe Institute for Advanced Study, Harvard University, Cambridge, MA 02138, USA.

## Abstract

Invertebrates constitute the majority of animal species and are critical for ecosystem functioning and services. Nonetheless, global invertebrate biodiversity patterns and their congruences with vertebrates remain largely unknown. We resolve the first high-resolution (~20-km) global diversity map for a major invertebrate clade, ants, using biodiversity informatics, range modeling, and machine learning to synthesize existing knowledge and predict the distribution of undiscovered diversity. We find that ants and different vertebrate groups have distinct features in their patterns of richness and rarity, underscoring the need to consider a diversity of taxa in conservation. However, despite their phylogenetic and physiological divergence, ant distributions are not highly anomalous relative to variation among vertebrate clades. Furthermore, our models predict that rarity centers largely overlap (78%), suggesting that general forces shape endemism patterns across taxa. This raises confidence that conservation of areas important for small-ranged vertebrates will benefit invertebrates while providing a “treasure map” to guide future discovery.

## Teaser

A high-resolution map of ant diversity allows an assessment of how well biodiversity centers overlap across taxa.

## INTRODUCTION

Thirty-five years ago, invertebrates were famously called “the little things that run the world,” (*1*) highlighting the importance of organisms that often remain in the background while vertebrates take center stage. Today, the critical roles of invertebrates for ecosystem functioning and services (*2*, *3*) are more widely recognized in both the public consciousness and in organized conservation efforts. However, we still lack an understanding of the patterns and dynamics of Earth’s invertebrate biodiversity, including basic questions such as which areas have the most species, which areas harbor concentrations of small-ranged species, and even whether there is a major global decline in insect biomass underway (*4*).

Without comprehensive, high-resolution geographic datasets on invertebrate groups, our knowledge of global biodiversity patterns is highly biased toward one branch of the tree of life: vertebrates (*5*). This vertebrate bias could undermine the effectiveness of area-based conservation efforts (*6*, *7*) even as these appear to be gaining momentum. It is possible that global-scale vertebrate biodiversity patterns are representative of invertebrate diversity patterns, but this potential remains mostly untested [but see (*8*)]. Recent studies on global species richness patterns of invertebrate groups suggest important divergences from vertebrates (*9*, *10*), but formal comparisons have not been made, and the distributions of small-ranged invertebrates—which are of critical importance to conservation—are nearly unknown.

In addressing these knowledge gaps, we face a fundamental data impediment. There are far fewer researchers and data collectors for invertebrates than for vertebrates (*11*, *12*), despite the former constituting orders of magnitude more species. Without a base of experts to consult for each species, we need to rely heavily on computational approaches to overcome several challenges in assembling a map of global diversity. First, we must synthesize information scattered in (often obscure) literature, museum collections, and specimen databases. Second, such data and metadata require vetting, updating, and interpretation. Third, we need a workflow that takes occurrence data for thousands of species spanning a broad spectrum of data size and quality and then returns reasonable estimates of these species’ distributions. Last, we need to account for different levels of research attention across the globe, resulting in geographic variation in field sampling intensity, the uneven taxonomic study of those samples, and variable progress in the continued improvement of taxonomic frameworks over time (*11*). Inventories are still incomplete for even the most conspicuous invertebrates in the best-studied regions (*13*). Thus, methods to estimate and account for these biases are needed, and ideally, these would also guide future inventory efforts toward efficient discovery (*14*).

Here, we address these challenges for ants and provide a uniquely comprehensive, high-resolution global biodiversity map for a major invertebrate taxon. Ants are ecologically dominant and economically important insects that play critical roles in ecosystems (*15*, *16*). Their known species richness is comparable to birds and mammals combined, and thus high enough to be informative while still tractable. They are also globally widespread (*17*, *18*) and abundant (*19*, *20*), and represent a good proxy for the diversity of other arthropod groups at both local and regional scales [e.g., (*21*, *22*)]. These traits, combined with recent data synthesis efforts (*23*, *24*), make them an attractive test case to assess congruence between invertebrate and vertebrate diversity centers.

We present the most comprehensive ant occurrence dataset to date and use a multifaceted informatics and modeling pipeline (fig. S1) to reconstruct a global biodiversity map at 10–arc min resolution (~20 km at the equator) encompassing nearly all described ant species. We use estimates of species ranges to calculate global maps of ant species richness (the number of species in an area) and rarity (richness weighted by range size to emphasize small-ranged species). Previous analyses of global invertebrate diversity either used point samples of alpha diversity to model geographic patterns of species richness (*9*, *17*, *25*) or were limited in resolution to large administrative regions (*10*, *18*), neither of which allow for both fine-scale richness and rarity estimates. Furthermore, we use machine learning models [Random Forest (*26*)] to account for the effects of sampling bias on the diversity maps and predict which areas may harbor hidden ant diversity. We then assess the congruence of biodiversity patterns for ants and vertebrates (amphibians, birds, mammals, and reptiles). The global distributions of different vertebrate taxa are themselves not fully congruent, as each has distinct features (*27*, *28*). Thus, our main goal is to determine whether biodiversity patterns for ants are comparably similar to those of vertebrate groups as these groups are to each other, or indeed highly divergent given their large phylogenetic, physiological, and ecological differences. Finally, we evaluate how well current protected areas capture important biodiversity centers.

## RESULTS AND DISCUSSION

Our data compilation (fig. S1) recovered 1,802,913 occurrence records for the native distributions of 15,463 valid species and subspecies. From these raw records, after data correction, georeferencing coordinates from existing locality metadata, error checking, and filtering out records with errors or high uncertainty, 1,479,293 records (representing 14,328 species) were used for analysis. This constitutes considerably more geographic coverage than previous global studies on invertebrates [e.g., this study: 159,061 unique georeferenced coordinates, mapped onto 47,385 unique 10–arc min grid cells (~20 km); as compared to earthworms, (*9*): 9212 unique sites and nematodes (*25*): 6759 samples, 1876 unique 30–arc sec grid cells (~1 km)]. These data were used to make range estimates with different methods based on data available for each species [buffered point(s): 5168 species; polygon based on alpha hull: 1554 species; or species distribution model (SDM) prediction within the alpha hull (after further spatially thinning occurrence records): 7606 species; see fig. S1]. As the uncertainty inherent in most of the occurrence data was on the order of 1 to 20 km, we performed our mapping and modeling at 10–arc min spatial resolution (~20 km at the equator). We estimated ant richness and rarity by aggregating species range estimates and defined centers of diversity as the top 10% of area for each.

### Global patterns of ant species richness and congruence with vertebrates

**Fig. 1.**
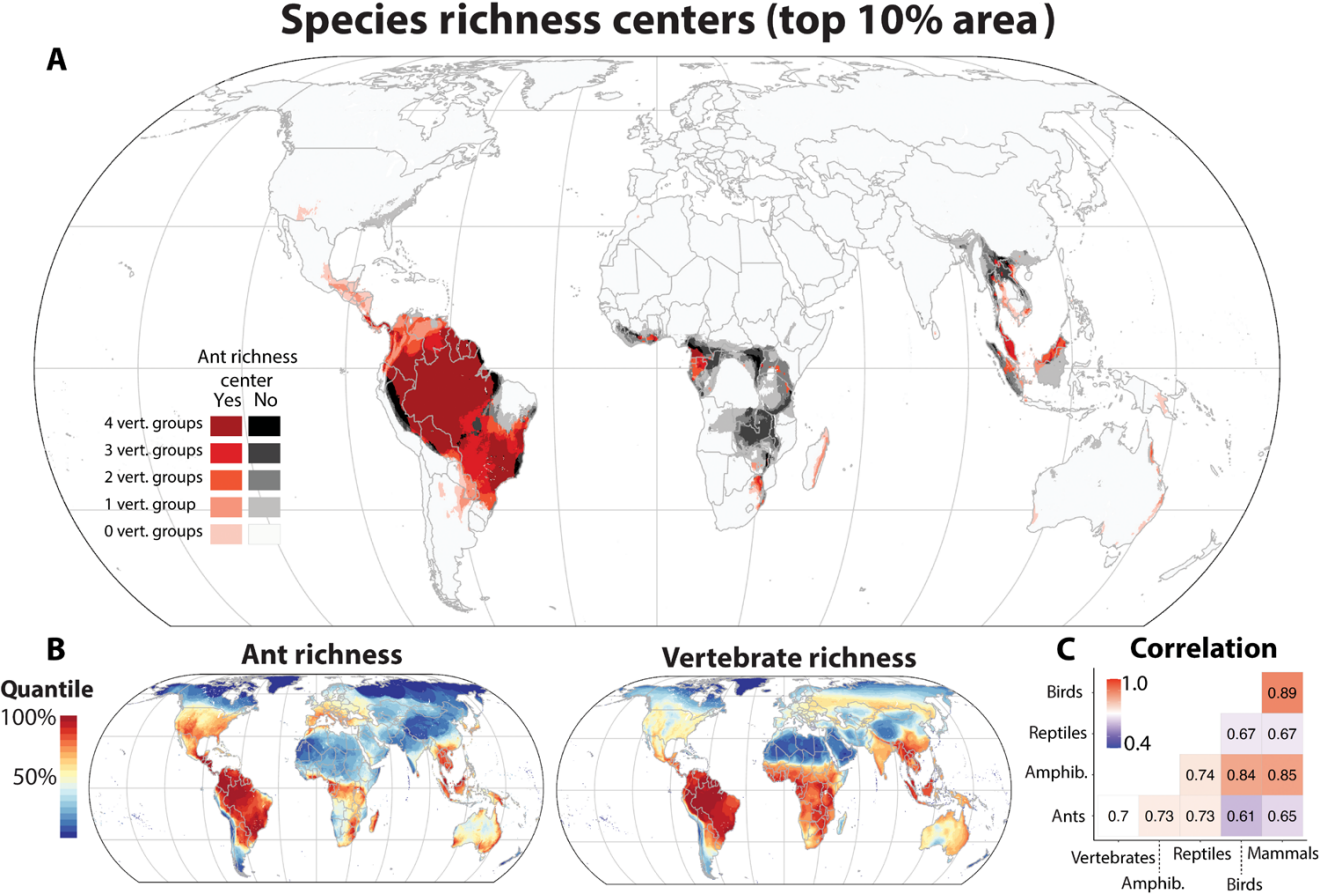
Global ant species richness patterns in comparison with terrestrial vertebrates. (**A**) Species richness centers (top 10% of area) for amphibians, birds, mammals, reptiles, and ants, indicating areas of congruence and incongruence of biodiversity centers across taxa. (**B**) Species richness maps based on stacking individual species range estimates for ants and vertebrates. (**C**) Spearman’s correlation matrix for grid cell–level species richness across taxa.

Ant species richness peaks in tropical regions (Fig. 1), consistent with previous work on ants (*17*, *18*, *29*) and many other groups (*30*), although inconsistent with global patterns for bees (*10*) and earthworms (*9*). Moreover, many of the regions identified within the tropics overlap with richness centers for vertebrate groups. Consensus species richness centers across taxa include the Amazon basin, the Atlantic Forest of Brazil, Mesoamerica, Central Africa, and Southeast Asia, although for the latter two regions, there is considerable finer-scale mismatch. The Afrotropics, in general, is also a relatively less rich region for ants than for vertebrates, particularly in the savanna and dry woodland areas of central and southern Africa. In contrast, the ant faunas in regions of New Guinea, Australia, and Madagascar are more species-rich relative to their global diversity than they are for vertebrates. Ant species richness (Fig. 1) is moderately correlated with richness of all vertebrates (Spearman’s rho = 0.70) and slightly less correlated on average than vertebrate groups are with each other (Spearman’s rho: ants-vertebrates mean = 0.68; vertebrates-vertebrates mean = 0.78; Fig. 1C and table S2). The overlap of richness centers between ants and vertebrates is also high and comparable in magnitude to the overlap of vertebrate groups with each other (ants-summed vertebrates = 0.72; ants-vertebrates mean = 0.68; vertebrates-vertebrates mean = 0.71; Fig. 1C and table S4).

We test the robustness of our richness estimates by exploring different methodological variations. First, we evaluate whether the species distribution modeling step [complexity-tuned Maxent models (*31*, *32*) predicted within the range polygon] has undue influence on the recovered richness centers, and we find that while these models contributed more detailed estimations of species ranges relative to univalue polygons, their effects on the location of richness centers are minimal (fig. S2). Second, we compare our richness estimates to a separate dataset [Global Ants Database (GLAD) (*24*)] of local community measurements of alpha diversity that (unlike our analysis) includes morphospecies. Although there is considerable variation in richness among local communities even on small spatial scales, the grid-cell prediction is both positively correlated and forms an upper bound for local measurements (i.e., local richness mostly does not exceed modeled regional richness). Third, we evaluate the robustness of our primary method of estimating and stacking species range estimates [e.g., (*33*)] using three alternate methods: (i) rarefaction and extrapolation of sampling curves, (ii) taxonomic surrogacy models (modeling species richness as a function of genus richness), and (iii) a climate-based macroecological model based on the GLAD data. In general, we find consistency between these methods and the range-stacking approach (fig. S3). However, we note that the only method that did not use the Global Ant Biodiversity Informatics (GABI) occurrence data (i.e. the macroecological model based on community data) did have the lowest correlations with all other methods (Spearman’s rho = ~0.6, compared to >0.8 for the other methods), although as a model of alpha diversity, it is the least comparable as local and regional diversity are not always correlated. To determine how robust our results were to changes in spatial resolution (*34*), we recalculated correlation of continuous values and overlap of diversity centers across varying spatial resolutions ranging from about 20 to 1000 km at the equator (10, 20, 50, 100, 200, and 500 arc min) and found little change in the broad-scale diversity patterns we observed (fig. S4).

### Global patterns of ant rarity and congruence with vertebrates

**Fig. 2.**
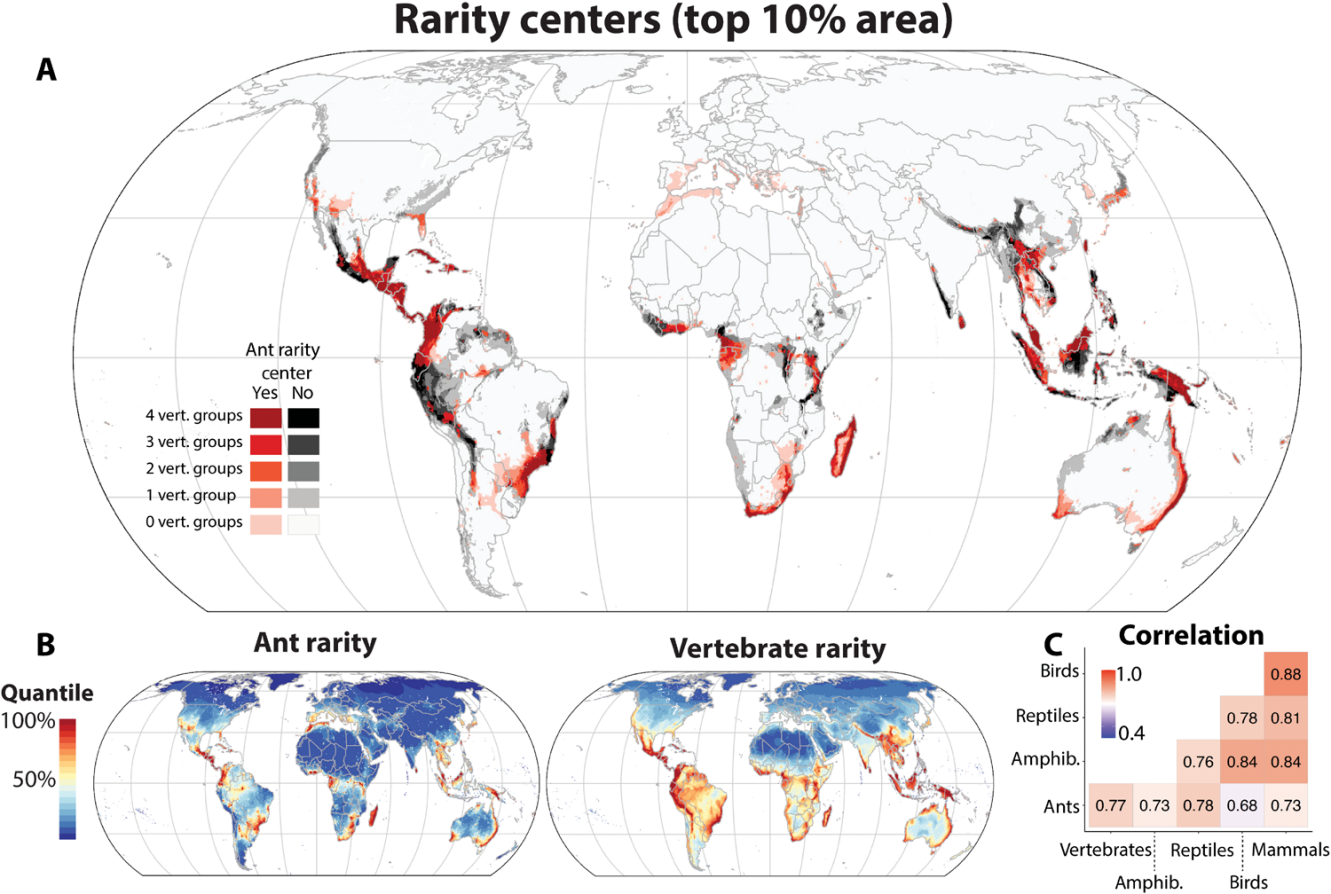
Global patterns of ant rarity and comparison with terrestrial vertebrates. (**A**) The concordance of different rarity (i.e., rarity-weighted richness, a metric indicating a concentration of small-ranged species) centers (top 10% of area) for amphibians, birds, mammals, reptiles, and ants. (**B**) Continuous rarity maps for ants and vertebrates. (**C**) Spearman’s correlation matrix for grid cell–level rarity across taxa.

Areas of high rarity (i.e., areas with many small-ranged species; Fig. 2) are used more frequently for conservation planning (along with assessments of threat status and economic, social, and local considerations) than areas of high species richness per se, as range-restricted species face increased threat of extinction (*35*, *36*). Moreover, richness patterns are known to be driven mostly by the distributions of widespread species (*37*). Around the globe, rarity centers (Fig. 2) are smaller, less contiguous, and more numerous than the fewer, larger centers observed for richness (Fig. 1). Ant rarity is correlated with vertebrate rarity (Spearman’s rho: ants-summed vertebrates = 0.77; ants-vertebrates mean = 0.73; vertebrates-vertebrates mean = 0.82; Fig. 2C and table S2), and although overlap fractions for diversity centers are slightly lower (ants-summed vertebrates = 0.53; ant-vertebrates mean = 0.48; vertebrates-vertebrates mean = 0.59; Fig. 2C and table S4), on a global scale, this level of overlap still represents a high level of agreement. Consensus areas of high rarity include Mesoamerica, South Africa, Madagascar, the southwest and eastern coasts of Australia, Southeast Asia, Sri Lanka, the western Congo, and New Guinea. However, regions of ant rarity appear to diverge from those of vertebrate rarity in several places: Small-ranged ant species are more prominent than small-ranged vertebrates in areas such as the southwestern United States, the Mediterranean basin, Japan, and the Korean peninsula (Fig. 2). Conversely, areas identified as rarity centers for vertebrates but missing for ants include several mountainous regions encompassing most of the Southern Tropical Andes, the Western Ghats, and the Himalayan region, among others. As with richness, we tested the effects of spatial resolution on the rarity estimates and did not find substantial sensitivity (fig. S5).

### Predicting richness and rarity accounting for sampling bias

While most biologists would not expect the global distributions of different taxonomic groups to be entirely congruent (*27*, *28*), it is important to evaluate whether apparent divergences are caused by real biological differences or reflect a geographically biased global inventory. In principle, sampling biases could increase or decrease correlations across taxa. We use “sampling” as shorthand for the entire process of field collection, specimen curation, taxonomic study, species description, and taxonomic revision over time, any of which could limit information on biodiversity in a region (as measured here by the density of ant occurrences for described species) and bias broader patterns.

We train Random Forest (*26*) models on our empirical ant estimates using potential predictor variables that reflect climate, topography, vertebrate richness/rarity, and global ant sampling intensity to predict how our estimates of richness and rarity may change with increased, and more spatially homogeneous, sampling around the globe. These models are (as expected) able to reproduce the existing ant empirical richness and rarity with high accuracy [complexity tuned with spatial cross-validation (*38*); see fig. S7], and we use these models to project changes to these patterns under a scenario of universally high sampling (i.e., the maximum sampling density observed for ants; fig. S6). For both richness and rarity, vertebrate patterns, sampling intensity, and several climate variables rank high on the basis of variable importance (fig. S7).

**Fig. 3.**
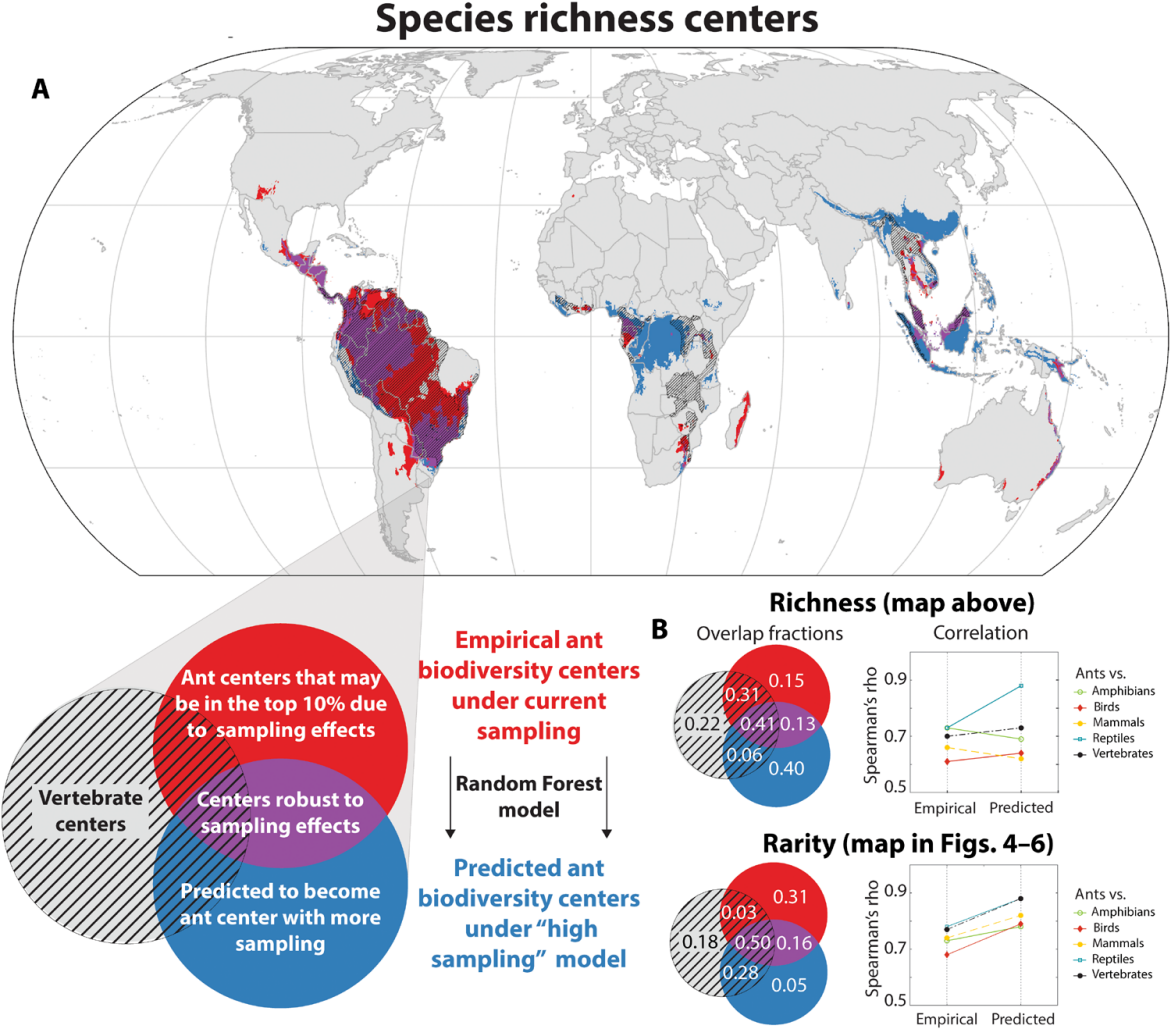
Machine learning predicts how increased sampling could change our understanding of ant richness and rarity centers. Random Forest models were trained to predict ant species richness and rarity values as a function of climate (7 vars.), topography, biogeographic realm, vertebrate biodiversity, and sampling density. We then used the models to predict (**A**) richness and rarity values under a “universal high sampling” scenario, revealing which areas may drop out of the top 10% with increased global sampling (red), which are robust to sampling (purple), and which centers are predicted to enter the top 10% with increased sampling (blue). The latter represents a treasure map indicating areas that should be prioritized for future sampling. The top 10% areas for vertebrates are indicated by hatched regions. (**B**) Overlap fractions for empirical and projected center designations for richness and rarity, and Spearman’s correlations continuous richness and rarity values.

**Fig. 4.**
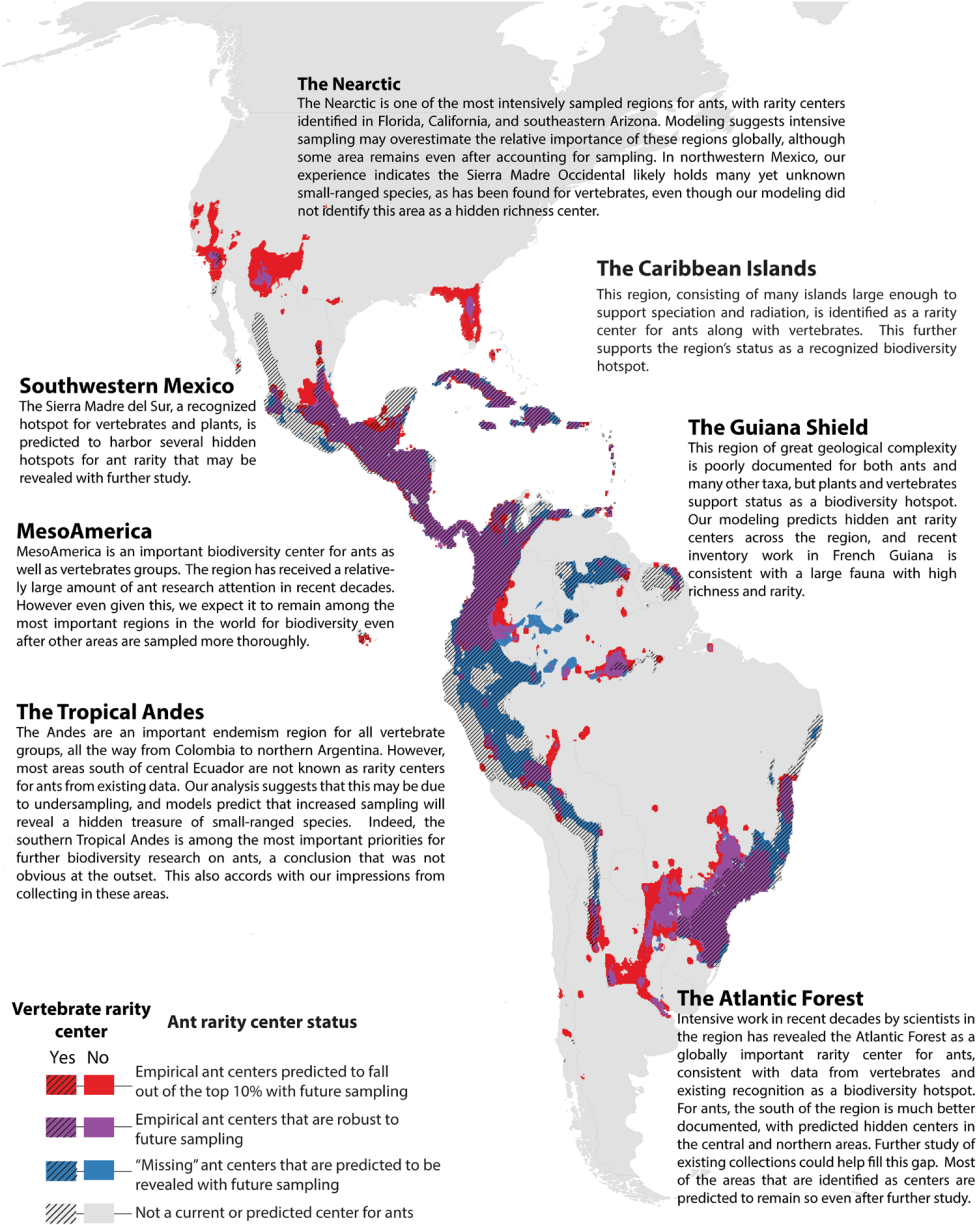
Empirical and predicted rarity centers of the Western Hemisphere. Rarity centers based on current knowledge and projected by a Random Forest model under a “universal high sampling” scenario. See Fig. 3 for more explanation.

**Fig. 5.**
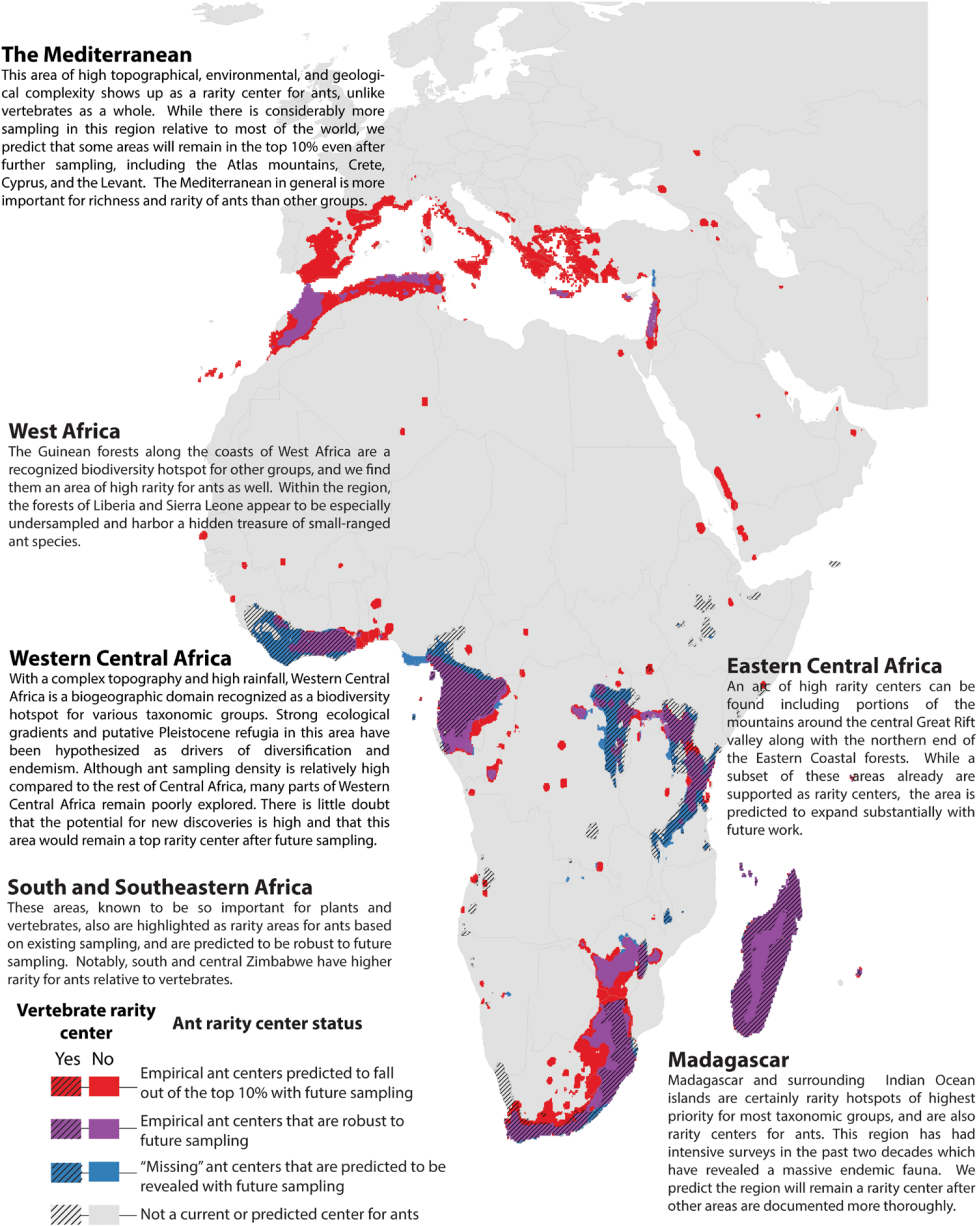
Empirical and predicted rarity centers of Europe, Africa, and West Asia. Rarity centers based on current knowledge and projected by a Random Forest model under a “universal high sampling” scenario. See Fig. 3 for more explanation.

**Fig. 6.**
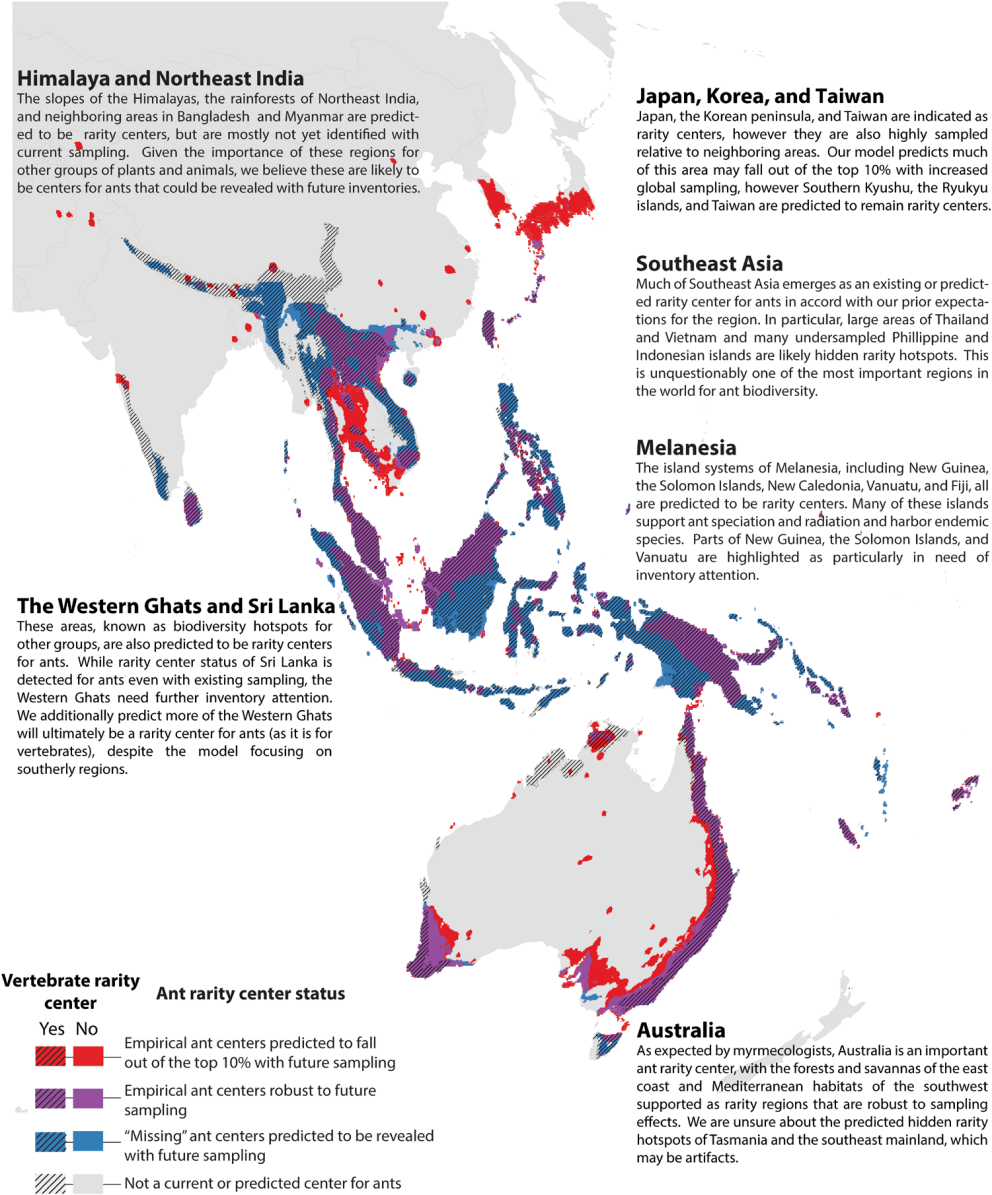
Empirical and predicted rarity centers of Eastern Asia and Oceania. Rarity centers based on current knowledge and projected by a Random Forest model under a “universal high sampling” scenario. See Fig. 3 for more explanation.

We find that 54% of the original center area for richness (Fig. 3) and 66% for rarity (Figs. 3 to 6) are robust to sampling effects (i.e., they remain in the top 10%). If future sampling targets those areas that currently have low sampling intensity, however, the locations of the remaining diversity center areas are predicted to change. Under this scenario, richness centers are predicted to expand in central Africa, Indonesia, southern China, and New Guinea. In general, accounting for sampling has divergent effects on richness and rarity. Our model predicts that additional sampling for ants will considerably reduce the overlap between vertebrate and ant richness centers [shifting from 72% for empirical versus 47% for modeled after future sampling; Fig. 3]. Moreover, while richness patterns are predicted to change to some degree, there is little effect on correlation of continuous richness values between ants and vertebrates (Spearman’s rho increases from 0.70 for empirical to 0.72 after correcting for sampling). The model also predicts that the existing global pattern of rarity centers will also be substantially altered with future sampling, but in this case, the diversity center areas are predicted to overlap more with those of vertebrates (53% for empirical versus 78% for modeled after future sampling; Figs. 3 to 6), and correlations overall will increase more substantially than for richness (Spearman’s rho increases from 0.78 for empirical to 0.88 after correcting for sampling). This shows that vertebrate rarity patterns are predictive of both known and yet undiscovered areas of high ant rarity.

### A “treasure map” for biodiversity

The model predictions for ant rarity centers not yet revealed by sampling represent a treasure map of hidden biodiversity, which can be used as a guide for future discovery of small-ranged species (Figs. 4 to 6). Such regions include the Southern Tropical Andes, the Western Ghats, much of Southeast Asia, and parts of New Guinea, all of which are rarity centers for vertebrates but not for ants based on current data. Many of the higher-latitude areas currently in the top 10% may drop out as the tropics become more thoroughly sampled. However, one notable area that is predicted to remain a biodiversity center for ants even with higher global sampling is the Mediterranean region, which is not in the top 10% for any vertebrate group. Further analysis and discussion of individual centers for each region are presented in Figs. 4 to 6.

### Protection status of biodiversity centers

**Fig. 7.**
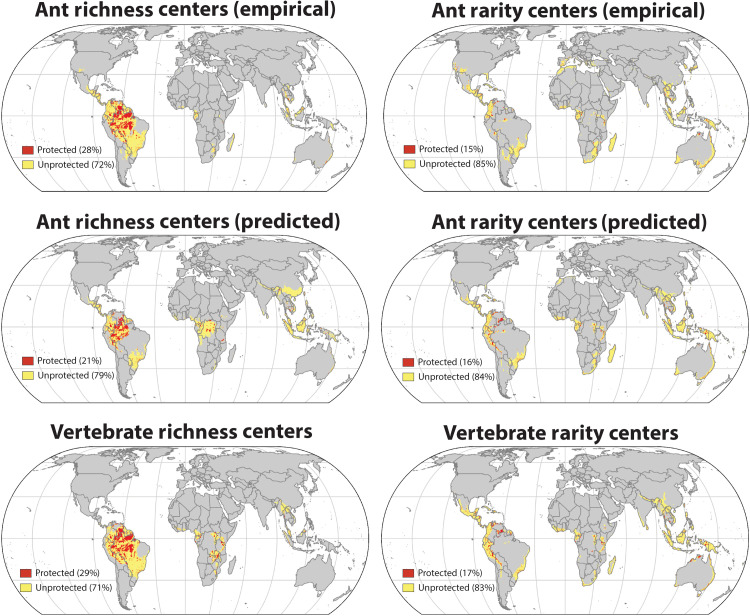
Global protection status of richness and rarity centers. Richness and rarity centers (top 10% of area) are overlaid with protected areas using data retrieved from the World Database of Protected Areas (protectedplanet.net) and processed. Biodiversity centers for ants based on current sampling (top row), predicted ant centers under universal high sampling (second row), and vertebrate centers (bottom row) are presented.

Large-scale biodiversity maps are not a panacea for conservation (*39*), but rather one component of a multiscale approach that integrates ecological, socioeconomic, geopolitical, and cultural factors (*40*). Our maps and highlighted areas considering biodiversity alone should not be considered global conservation priorities per se, but they do provide foundational knowledge that should help guide biodiversity conservation to better incorporate insects. Moreover, a global perspective allows us to zoom out and take stock of how well current protected areas currently overlap with biodiversity centers. While our analysis raises confidence that many important biodiversity centers are shared across widely divergent taxa, we also find that regardless of metric or taxon, only a modest fraction of these areas currently have protection (15 to 29%; Fig. 7), with rarity centers among the least likely to have preservation status.

### Future directions

While we built a workflow to overcome inherent data challenges, each step of the process required methods and choices that could undoubtedly be improved with further research on best practices relative to the specific goal of generating a biodiversity map. As we work toward developing comprehensive maps for a greater diversity of taxonomic groups, the data impediments for other taxa will more likely resemble the challenges presented by ants than the special cases of highly studied vertebrates. Thus, we need more research on optimized methods and workflows to apply across the tree of life. Data limitation should not be an excuse to avoid understudied groups or dismiss their relevance, but rather a reason to focus our ingenuity on meeting an informatics challenge.

Second, this analysis sets the stage for a new round of investigation of the mechanisms underlying both ant biodiversity patterns and cross-taxon congruence. For example, ant biodiversity patterns are most congruent with those of reptiles, echoing a similar finding for the global spread of introduced species (*41*), but the reasons for this correlation are unknown. Moreover, although global plant datasets at a comparable resolution were not available to include in this study, illuminating the level of geographic covariation between plant and insect diversity is a critically important goal for both basic science and conservation.

Last, while our models aim to fill geographic gaps in knowledge, they are at best a temporary stopgap while we continue our global inventory. Models are hypotheses, and predictions of hidden diversity must be tested with future targeted inventory efforts for specific areas. Our analyses highlight how focused efforts can greatly increase knowledge of regional biodiversity; for example, Mesoamerica, Madagascar, Colombia, and the Atlantic Forest of Brazil are now robustly documented as ant biodiversity centers, largely due to vigorous research activity in these areas in recent years. We identify regions that should be special priorities for new inventory work (encompassing new field sampling, taxonomic analysis of existing collections, and revision of earlier taxonomic work), underscoring the need to expand taxonomic and scientific capacity in those regions. With that said, nearly all areas are understudied for ants to some degree, and thus, inventory work should be encouraged everywhere.

Our study provides the first global, high-resolution map for any major insect group encompassing all described species. While it was possible a priori that ants could be highly divergent in their patterns from vertebrates, we found substantial—albeit imperfect—congruence across these distant taxa. Ants are only modestly less correlated with vertebrate groups than vertebrate groups are with each other. These results for ants imply that conservation efforts aimed at vertebrates are likely to capture a diversity of insects as well, and these correlations exceed expectations from shared responses to general climate gradients (e.g., higher diversity in the humid tropics). The predictive value of vertebrate biodiversity patterns is particularly notable for rarity—the most critical metric for conservation—as our Random Forest models predict that many (but not all) of the differences between ant and vertebrate centers can be attributed to sampling biases. This suggests that ecological and historical forces shape endemism patterns in ways that can be generalized across divergent taxa. Vertebrate patterns are the most informative variables (along with sampling density) in our models, being more important than climate or biogeographic realm.

That said, the fact that groups are correlated does not imply they are interchangeable. Ant patterns have distinct features, as do each of the vertebrate groups, and it is likely these apparent idiosyncrasies are shared by many other taxa that are not yet represented in global analyses. Furthermore, birds and mammals, the taxa that usually receive preferential conservation attention (*5*), are tightly correlated with each other but less correlated with the ectothermic groups of amphibians, reptiles, and ants. Thus, even as some generalities emerge, there is a critical need to increase the diversity of taxa informing our understanding of global biodiversity.

To protect species, we need to know where they are, but global inventories for most invertebrate groups will take decades or more to complete. Our analysis shows that informatics and modeling approaches provide an alternate route to resolving a provisional biodiversity map for data-deficient groups. While our analysis raises confidence that many important biodiversity centers are shared across widely divergent taxa, we also find that only a modest fraction of these areas are protected, highlighting the need for bold and rapid action to protect Earth’s biodiversity.

## MATERIALS AND METHODS

### Experimental design

The overall analysis workflow (fig. S1) has the following steps: (i) compile raw data from diverse sources and harmonize taxonomy; (ii) clean, vet, and georeference the data; (iii) use point occurrence data to model and estimate species distributions; (iv) aggregate species distributions into composite biodiversity maps and identify species richness and rarity centers; (v) model and predict how diversity centers will change with future sampling, including identification of areas with undiscovered diversity; (vi) compare ant biodiversity centers with vertebrate centers; and (vii) assess the degree to which biodiversity centers are captured in existing protected areas. All programming code, occurrence data (pre- and postprocessing), model results, and diversity estimates can be found in data S1 (https://doi.org/10.5061/dryad.wstqjq2pp).

### Data compilation

The GABI database is intended to consolidate and curate ant geographic biodiversity data in a single place, synthesizing data from literature, online databases, museum databases, and personal collections (*23*) (see table S1 for detailed data sources and data S1 for the full dataset). At the time of this study (data downloaded 14 July 2020), it is composed of 2,466,704 total records, but only 1,802,913 (73%) have a valid species name (following species nomenclature from AntCat.org (*42*)) and are not dubious or exotic records. We put these records through a data-cleaning, georeferencing, and optimization pipeline. Of these, 766,854 records we compiled from the literature, 1,177,653 records from 81 public databases [the largest component being 420,251 records from AntWeb (version 8.66) (*43*)], and 42,084 records from personal collections and communications, representing 15,463 known ant species and subspecies (not including dubious or exotic records). The compilation workflow is summarized in fig. S1; more details on the methods for data compilation and databasing are described in a previous publication (*23*), and the data can be viewed through https://antmaps.org/ (*44*). Of these raw records, most lack georeferenced occurrence points, and many existing point records have errors. While previous analyses using earlier versions of this dataset assigned these records manually to large administrative areas (e.g., states, countries) for analysis [e.g. (*18*, *41*)], here we used an informatics pipeline to clean and convert these raw records into an extensive georeferenced dataset, which we then used for downstream range modeling and diversity mapping.

### Data cleaning, georeferencing, and optimization

Species occurrence data are typically fraught with errors, missing fields, and various biases that necessitate cleaning and verification steps before it is used in analyses (*45*, *46*). Although several established tools exist for processing occurrence data [e.g., (*47*)], including some introduced quite recently (*48*, *49*), most focus on online biodiversity databases, and the diverse nature of occurrence data problems often leads to low effectiveness of any one tool when applied to a new dataset (*50*). These issues are compounded for datasets like GABI with a global extent, which include areas of the world poorly represented in locality-validation databases and records that originate from varied data sources. We initially experimented with single tools to validate and clean the GABI occurrence data, but found this strategy resulted in many inaccuracies, especially for regions outside North America, Europe, or Australia. As GABI has over 2 million data records, it proved infeasible to manually inspect each record and correct inaccuracies. Geocoding tools, such as those offered by Google and implemented through Python or R packages, are not used often in ecological and evolutionary studies, but they can help fix a variety of spatial errors and suggest correct positioning of coordinates. We thus opted to use a combination of functionality from existing occurrence data cleaning tools, geocoding tools, and custom scripts that automate cleaning and validation procedures existing tools did not offer and then make manual fixes when necessary to optimize data accuracy (fig. S1).

Our custom geoprocessing pipeline (i) converts raw text and numeric data gathered from the literature or specimen databases into georeferenced occurrence points and (ii) cross-checks point accuracy using a variety of methods (numbers here refer to R and Python scripts and can be found in data S1, https://doi.org/10.5061/dryad.wstqjq2pp). We first retrieved 1,802,913 raw ant occurrence records from the GABI database (downloaded 14 July 2020), of which only 1,062,720 records had coordinate information [1]. To prepare the data for geocoding, we pooled duplicate localities based on locality or archipelago information from multiple fields and latitude/longitude coordinates (when available) rounded to four decimals (~11 m; [2]), ensured proper encoding for numeric fields [3], ensured coordinates did not have obvious errors in location (e.g., outside of ±180°) or formatting [4], corrected character-encoding errors for locality fields (Python package *ftfy* (*51*); [5]), measured the fuzzy distance between original and corrected country names (Python package *FuzzyWuzzy* (*52*); [5]), and detected additional coordinate information by scanning text in other fields [6]. We then addressed any remaining character-encoding errors for locality fields and confirmed country attributions by checking against global locality-validation databases [GeoNames (<https://geonames.org/>), United Nations Trade Statistic Country Code (<https://unstats.un.org/unsd/tradekb/Knowledgebase/Country-Code>), Geo-Locate (<http://geo-locate.org/>), and Google geocoding returns] to generate a standardized locality string [7], and, finally, produced the geocoding dataset representing all the unique localities in the data [8]. We conducted reverse geocoding (finding locality information based on available coordinates) and forward geocoding (finding coordinates based on locality information) on the cleaned dataset via the Google Geocoding API (*53*) using the geocoder Python package (*54*) (9) and then cleaned and parsed the geocoding return data, assigned levels of spatial precision [10], and integrated these data with the original database [11]. We filtered out all geocoded results with precision radius higher than 100 km. This radius refers to the area interpreted by the Google geocoder, returned as the “viewport,” and is calculated using the geodesic distance of the northeast and southwest points of the viewport. Essentially, we used the viewport to constrain geocoded regions to within a 100-km radius to limit centroids that would otherwise represent a very large region. For the 10–arc min bioclimatic raster data we used in our analysis, this would represent a potential error (at its worst) of ~5 pixels from the center in each direction at the equator. We selected which records to retain using a decision tree based on agreements between locations (countries and administrative regions) and pairs of records (original and geocoded, where original records are prioritized) [12]. The optimal records were then run through the CoordinateCleaner R package (*48*), which calculates metrics to judge the validity of the point data. We used this package to remove records that were judged to be polygon centroids and then made manual edits based on identified outliers for final locational corrections as many of the flags identified by the package were not applicable for ants and needed expert opinions to verify. This process assigned optimal coordinates derived from existing record metadata for 988,331 records (112,256 unique coordinates) and generated optimal coordinates for 490,962 geocoded records (48,675 unique coordinates), resulting in an optimized georeferenced dataset of 1,479,293 total records. No geocoded records were retained in the optimized dataset with a precision radius greater than 100 km, and most (82%) of these records have a spatial precision under 20 km.

### Species range estimates

For each species, we made polygonal range estimates and additionally used species distribution models (SDMs) to estimate suitability within these shapes for species with sufficient data (fig. S1). We first estimated ranges with alpha hulls [R package alphahull (*55*) with alpha value 15] for species with ≥3 occurrence localities (9156 species; 4 species used alpha shapes because of issues fitting alpha hulls) and with buffered points (30 km) for species with <3 occurrence localities (5168 species). We decided on an alpha value of 15 for alpha hull polygonal range estimates (for species with <5 occurrence localities) and SDM study extents (for species with ≥5 occurrence localities), as it resulted in ecologically realistic range shapes over the broad spectrum of point patterns for the ant occurrence data. We buffered alpha hulls and points by 30 km to account for spatial uncertainty, using the R package geobuffer (*56*) to make geodesic buffers for point data. As geodesic buffering for polygons was not possible in R and difficult to implement otherwise, we used the package rgeos (*57*) to make Euclidean buffers for polygonal data after projecting the data to the World Behrmann equal-area projection (as it allowed for some tools to draw geometry past the prime meridian instead of cutting it off using the “+over” specification in the coordinate reference system definition), and then projected back to the original World Geodetic System (WGS) 1984 geographic coordinate system. For species with ≥5 occurrence localities (7606 species), we first spatially thinned occurrences by 10 km [R package spThin (*58*)] to reduce the effects of sampling bias and then built SDMs to predict suitability within their study extent, which was defined by their buffered alpha hull range estimate. We used the presence-background machine learning algorithm Maxent 3.4.1, which fits a relationship between occurrence localities and environmental variables constrained to best match the background environmental distribution (*32*). For predictor variables, we used 19 global bioclimatic variables at 10–arc min resolution (approximately 20 km at the equator) from the WorldClim 2.0 dataset (*59*) representing long-term annual trends, seasonality, and extremes that are relevant to describing the spatial patterns of species’ ranges. The resolution was chosen to reflect the precision of the compiled and georeferenced data. From these rasters, we removed areas corresponding to inland water bodies (from <https://naturalearthdata.com>) and then masked them to each species’ alpha hull range estimate for modeling using the R package raster (*60*). As inspections of the ant occurrence data revealed strong signals of sampling bias for multiple species even after spatial thinning, we sampled background localities for each SDM with the same bias inherent in the occurrence data using a sampling density grid (*61*). To make this grid, we calculated the kernel density of the occurrence localities for all ant species in the GABI database across the globe [fig. S6; ArcGIS Pro 2.6.0 (*62*)] and removed inland water bodies. Then, after transforming these density values to a range between 0 and 1, we used them as proxies for sampling effort and probabilistically sampled 10,000 background points for each species. Species with smaller study extents (<10,000 grid cells) were assigned full background samples (i.e., all grid cells) to avoid using too few background localities for model training.

For each species with sufficient data after spatial thinning (≥5 occurrence localities, *n* = 7606), we built a range of SDMs with differing levels of complexity and selected optimal models per species using sequential selection criteria based on cross-validation evaluations. Maxent has two main hyperparameters for managing model complexity: Feature classes control the shape of the model response, and regularization multipliers control how much complexity is penalized. As default settings in Maxent can result in overfit models, we built SDMs for each species with different combinations of feature classes [linear (L) linear-quadratic (LQ), hinge (H), and linear-quadratic-hinge (LQH)] and regularization multiplier values [1 (default: low penalization) to 5 (higher penalization)] to evaluate models with a range of different complexities, from simple linear relationships to complex ones with curvilinear fits or splines (*63*). In essence, the addition of complex feature classes (here, Q and H) adds potential complexity to the model, but increasing regularization will result in the increasing removal of both predictor variable features (i.e., quadratic or hinge fits) and the variables themselves [i.e., coefficients can be reduced to zero, dropping the variable from the model (*32*)]. We used the R package ENMeval 2.0.0 (*31*) to iteratively construct models with all combinations of these settings and evaluated models using *k*-fold cross-validation on withheld data: leave-one-out (i.e., delete-one jackknife) for species with <25 occurrence localities [as this method maximizes sample size of validation data (*64*)], and random (*k* = 5) for all others. We did not use spatial cross-validation for evaluation, as our aim was simply to choose the models that best predicted current data and not to transfer models to different areas or time periods (*38*). We used the following sequential criteria for model selection based on both threshold-dependent and threshold-independent performance metrics (*63*, *65*). First, we filtered out all models without nonzero coefficients (possible because high regularization can reduce all predictor variable coefficients to zero). Next, we filtered out models that performed poorly (≤0 or NA) for the Continuous Boyce Index [R package ecospat (*66*)] calculated on the full dataset, as positive values indicate that the model’s predictions are consistent with the distribution of occurrences (*67*). We chose to calculate the Continuous Boyce Index on the full dataset as a standard measure across all species as opposed to on validation data because for many species, the validation scores were NA because of low sample size for partitions. Of the remaining models, we first selected those with the lowest 10 percentile omission rate (the proportion of validation occurrences not predicted by the model after thresholding the prediction by the 10-percentile suitability value, which is more conservative than using the lowest suitability value for thresholding) and then broke ties by choosing the model with the highest validation area under the ROC curve (AUC) (*63*). Although calculating AUC with presence-background models is problematic when models with different training data or study extents are compared, relative comparisons for the same species and model settings are valid (*68*). We did not filter out models with values of AUC at or below 0.5 because although such scores may indicate an ability to discriminate between occurrences and background localities no better than random (*69*), this only holds true when absence data are used, and the particular value at which performance can be called poor is unknown when using background data (*70*). We note that in preliminary tests, we experimented with choosing optimal models using the Continuous Boyce Index as an alternative performance metric to validation AUC for this step in the sequential criteria but found that the result was NA for many low-data species and that the final richness predictions were very similar regardless of this choice—we thus chose to use validation AUC. If ties remained, we chose the model with the lowest number of nonzero coefficients to prioritize simpler models. Across modeled species, 33% (*n* = 2519) had minimum omission rate values that were unique, while 54% (4111) used validation AUC to break ties in minimum omission rate, and the remaining ones had ties in AUC that were broken by picking the model with the minimum number of nonzero model coefficients. We then used these models to predict suitability over each species’ alpha hull range estimate using Maxent’s “cloglog” transformation (*32*), which predicts a continuous scale between 0 and 1. As many ant occurrence records do not include consistent information for sampling date, and as article publication date can be a poor proxy for date of collection, our dataset is not temporally resolved. But although, the boundaries of range estimates based on older records may miss recent range shifts, the impact on global-scale patterns should be modest.

### Species richness and rarity calculations

We then stacked (i.e., combined) the species range estimates to produce maps of two key biodiversity metrics: species richness and rarity-weighted richness (*71*) (fig. S1; henceforth rarity). Species richness, the number of species present in an area (*72*), is a fundamental variable for ecology and conservation. However, because geographic variation in species richness is dominated by widespread species rather than those with small ranges, examining species richness alone will miss key areas of diversity that should be priorities for conservation attention (*73*). In contrast, rarity reflects the presence or absence of many small-ranged species in an area, with high values indicating concentration of biodiversity unique to a region.

We overlaid the species-level range estimates (polygonal and SDM-derived) to estimate global species richness and rarity patterns for ants (fig. S1). The richness of cell *i* was calculated as *s_i_* = sum(*w_j_*), where the weight for species *j* (*w_j_*) was either 1 (for polygonal range estimates) or the continuous Maxent cloglog prediction (*74*). As low-data species tend to have restricted ranges, there should be little, if any, associated bias in favor of these species in richness estimates. In calculations of rarity, species are often weighted by the inverse of range size, or *r_i_* = 1/(*a_j_*) for cell *i*, where *a_j_* is the area of the range of species *j*. However, this weighting across species is arbitrary, and we found that species with a single locality dominate the calculation. Although species truly confined to a single locality would certainly be of high interest for conservation, in practice, most such data are a product of “sampling islands” that underestimate the range extents of species found there. As a result, rarity can be locally overestimated, leading to many small and isolated diversity centers. To adjust the weighting across species, we added a constant *c* to make the rarity calculation *r_i_* = 1/(*a_j_* + *c*), which moderates the decline of the weight with increasing range size. This results in more balanced estimates between species with small and large ranges, reducing the dominance of single localities and thus removing some apparent sampling islands on the map. In experimenting with the data, we found that a value of *c* = 60,000 km^2^ (corresponding to a circular range with radius 138 km) was sufficient to “denoise” the plot without losing important smaller rarity centers, and we used this for the main analysis. We then projected each diversity estimate from its original geographic projection (WGS 1984) to the equal-area Eckert IV projected coordinate system (with bilinear resampling to the maximum value to ensure that maximum diversity per cell did not change) for mapping and further analysis.

### Comparison of richness estimates to community-level point observations

As a test of the performance of our ant richness estimates, we used simple linear models to determine how well our grid cell richness corresponds to maximum observed community richness from a mostly independent ant community database. While regional richness is not necessarily expected to match community richness, it should form an upper bound and be correlated. The Global Ants Database (GLAD) (downloaded 20 February 2020) consists of over 50,000 ant occurrence records (>2300 unique localities) from over 200 community ecology studies around the world (*24*). The GLAD database partially overlaps with the GABI database, in that occurrence records from community surveys may have been represented in published literature or specimen databases that were entered into GABI. However, GLAD also contains many unpublished datasets and, importantly, includes morphospecies information in richness measurements. As the range-modeling approach using GABI point occurrence data can only be performed with described species (because morphospecies cannot be matched across different studies or localities), it allows us to check whether our grid-cell estimates are much too low because of a low fraction of described species in some areas. We used the existing fields to subset an analysis dataset consisting only of native species found in undisturbed areas, used the corrected versions of species names, and retained the identities of morphospecies by giving them unique names linked to the study that identified them. We also removed all occurrences of “?” from species names, assuming that the presumed identifications were correct. We then associated each GLAD record with the corresponding grid cell from the GABI prediction (10–arc min resolution) and calculated richness per grid cell by study to avoid summing morphospecies across studies. When more than one study existed for a grid cell, we kept the highest observed GLAD richness per equal area grid cell for a range of increasingly coarser spatial resolutions (10, 20, 50, 100, 200, and 500 arc min) and matched these values to GABI-estimated richness values at the same cell. We then ran a simple linear model and calculated the coefficient of determination (*R*^2^) for each resolution (figs. S4 and S5). This methodology is similar to the approach used by Ballesteros-Mejia *et al.* (*75*), who validated their modeled continental-scale richness patterns for sphingid moths based on the observed richness at multiple “well-sampled locations.”

We found that the correlation between GABI and GLAD richness was, in general, relatively high (fig. S3). In line with expectations, correlation increased with coarser resolution, from 0.337 to 0.460, although *R*^2^ initially decreased slightly. The *R*^2^ values were as follows: 10 arc min, 0.337; 20 arc min, 0.336; 50 arc min, 0.339; 100 arc min, 0.355; 200 arc min, 0.404; and 500 arc min, 0.460. For all correlation analyses, all non-NA grid cell values were used, including both zero and nonzero values, and Antarctica was excluded.

### Alternative methods to estimate species richness

In addition, we determined that our ant richness estimates were mostly robust to alternative methods: rarefaction and extrapolation sampling curves, genus surrogacy models, and macroecological models based on climatic variables and community data from GLAD (fig. S3).

#### Richness from rarefaction/extrapolation moving window

We estimated richness using a rarefaction/extrapolation approach by calculating Hill numbers (*76*) (*q* = 0) based on the preprocessed GABI occurrence data (before removing point duplicates and spatial thinning for modeling). We used the R package iNEXT (*77*) to calculate the observed and estimated richness, as well as sampling completeness, for each grid cell based on the GABI occurrence data within a moving window (fig. S3). After experimenting with several different sizes, we decided to use 10–arc min grid cell windows of 60 × 60 (i.e., 10° × 10°) to balance the smoothness of the mapped result with an appropriate spatial resolution to visualize global patterns. We summarized the GABI occurrences for all cells within each window as a frequency table that represents the number of occurrences per grid cell for each species (i.e., “incidences”) and then input these frequencies and the total number of cells as sampling units into the iNEXT() function with the “incidence_freq” setting for data type. Although iNEXT does not use occurrence-based frequencies to calculate species richness, this parameterization returns a single estimate for the entire window instead of individual ones per grid cell, and so was more appropriate for our purposes. To avoid unreliable estimates, we followed a similar methodology to Kusumoto *et al.* (*78*) by omitting calculations for windows with few grid cells containing species incidences (in our case, <2) or those with as many singletons as there were total incidences. Unlike Kusumoto *et al.* (*78*), we did not apply a constraint to the number of species within a window as some areas had low taxonomic diversity but sufficient sampling.

#### Richness from genus surrogacy

As an alternative method for estimating richness, we used the “higher taxon surrogacy” approach (*79*) by modeling species richness as a function of genus richness for each set of grid cell predictions, with the assumption that species richness is more undersampled than genus richness. As ants are undersampled in general, particularly in certain regions of the globe, modeling the mean of richness would likely be an underestimate, and thus, we used quantile regression to model the upper bound. We estimated genus-level richness by first aggregating the GABI species occurrence data to the genus level, then applying the same methodology we used to estimate richness at the species level (i.e., stacking polygonal range estimates and SDM predictions). Aside from the necessity for the response to be monotonically increasing and convex in shape, we lacked a theoretical basis for specifying any particular parametric model for this relationship. We thus fit nonparametric additive regression models with the quantile (*tau*) set at 0.9 and additionally with constraints “increasing” and “convex” using the R package quantreg (*80*). We fit separate models for each biogeographic realm (*81*) with our estimates of species richness as the response variable and genus richness as the sole predictor variable, exploring a range of very small to relatively large lambda values for penalizing complexity (0.01, 0.1, 1, 2, 3, 4, 5, 6, 7, 8, 9, 10). For each realm, we selected the model with the lowest Akaike information criterion (AIC) value. When multiple models had delta AIC values (model AIC − minimum AIC across all models) equal to or less than 2, we chose the simplest model from this subset (i.e., the model with the minimum lambda value). We combined the predictions for each biogeographic realm to make a global map of richness estimates based on genus surrogacy (fig. S3).

Responses for 0.9 quantile models were all curvilinear with varying degrees of complexity. We also fit 0.5 quantile models with the same settings for reference. Realms with a high number of occurrence records resulted in low optimal lambda values, indicating the need for higher complexity to model more complex responses. The lambda values per realm were, from smallest to largest: Afrotropical, 0.01; Australian, 0.01; Nearctic, 0.01; Palearctic, 0.01; Saharo-Arabian, 0.01; Neotropical, 2; Madagascan, 4; Sino-Japanese, 6; Oriental, 7; Oceania, 10; and Panamanian, 10. In general, responses for both quantiles (0.9 and 0.5) remained similar for the full ranges of genus richness, with some exceptions: Oceania, Nearctic, and Panamanian had 0.9 quantile responses that diverged considerably higher for high genus richness values.

#### Macroecological model using GLAD community database

For the macroecological modeling approach, we took advantage of the GLAD (*24*) dataset to model ant species richness based on environmental variables. Using the methodology explained in ‘‘Comparison of richness estimates to community-level point observations,’’ we assigned maximum GLAD richness to 10–arc min grid cells. This resulted in 268 unique grid cells with maximum GLAD richness estimates with global representation (Africa, 28; Asia, 38; Europe, 40; North America, 43; Oceania, 46; and South America, 73). We modeled GLAD ant richness as a function of bioclimatic and biogeographic predictor variables. We selected a subset of the original 19 bioclimatic variables from WorldClim 2.0 (*59*) with low collinearity [variance inflation factor less than 3 (*82*)] using the R package *usdm* (*83*). We used a conservative threshold as regression models without regularization are sensitive to dependence between predictor variables caused by high collinearity (*84*). The six variables we retained for modeling were mean diurnal range (bio2), mean temperature of wettest quarter (bio8), mean temperature of driest quarter (bio9), precipitation seasonality (bio15), precipitation of warmest quarter (bio18), and precipitation of coldest quarter (bio19). We also used a dataset of global biogeographic realms based on vertebrate distributions and phylogenies (*81*). We first fit a generalized linear model with Poisson error distribution and log link function with the bioclimatic and biogeographic predictor variables, but as this model showed overdispersion, we then fit all subsequent models with a negative binomial error distribution. We performed an exhaustive model selection procedure with the R package *MuMIn* (*85*) and used the sample-size corrected Akaike information criterion (AICc) to determine the optimal combination of predictor variables. The optimal model included all the input predictor variables, and all other models were suboptimal as they had delta AICc >2. As some predictor variable ranges for the globe included values outside those used for model training, we made “clamped” predictions (fig. S3) that used modified versions of the original predictor variables that were constrained to their respective ranges from the training data (*86*) using the function clamp.vars() in *ENMeval* 2.0.0 (*31*). Thus, our predictions do not make extrapolations beyond the bounds of the training data. Ultimately, there were few major differences between the unclamped and clamped predictions that extended mainly to very small areas. We do note that some regions that our range-stacking approach found were richness centers for ants, such as the African Mediterranean, Central Africa, Madagascar, and parts of Southeast Asia, have relatively poor coverage in the GLAD database, which can perhaps explain why this approach had the least correlation with the other methods. Of these regions, only Central Africa had a high richness prediction that was generally congruent with the other methods.

### Terrestrial vertebrate data

We compared the stacked estimates of ant richness and rarity to corresponding maps we made using existing range datasets of terrestrial vertebrates: amphibians, birds, mammals, reptiles, and these groups combined (henceforth “vertebrates”). These data come from different sources: International Union for Conservation of Nature (IUCN) range maps for amphibians and mammals (2010) and BirdLife International NatureServe breeding ranges for birds (2011) are described in Jenkins *et al.* (*27*) (2013), and range maps for reptiles were developed by Roll *et al.* (*28*). To match our methodology for GABI polygonal range estimates, we overlaid the polygonal range estimates for each taxon and summed the overlapping polygons for each 10–arc min grid cell. Although we buffered range estimates for ants (30 km) to account for geographic uncertainty, we chose not to buffer those for vertebrates. As the vertebrate range estimates were delineated with expert knowledge, buffering would likely lead to overestimation, as well as change their shapes from those previously reported and used for analysis. For comparisons with ant diversity maps, all maps for vertebrates were also projected to the equal-area Eckert IV projected coordinate system, and Spearman’s correlations among grid cells were calculated to give an overall estimate of congruence.

### Diversity center calculations

We calculated diversity centers for the richness and rarity maps of both ants and vertebrates to make comparisons of such areas among taxa. Centers were defined for each diversity metric as the top 10% quantile after excluding Antarctica (table S3). Other studies have used smaller thresholds, such as 2.5% (*87*), 5% (*27*), or a range from 2.5 to 10% (*28*), but these focused on more well-known terrestrial vertebrates. As this is the first study of this scale for insects, we decided to use a less conservative threshold for diversity centers to identify a broader selection of areas of conservation importance. We made comparisons between the richness and rarity centers for ants and each vertebrate group, including the vertebrates combined (Figs. 1 and 2).

### Tests of sensitivity to spatial resolution

As our richness and rarity estimates for ants were conducted at the relatively fine resolution of 10 arc min at a global scale, we sought to determine how robust the observed diversity patterns were to coarser spatial grains. Compared with richness estimates derived from range maps, those derived from SDMs have been shown to reveal greater heterogeneity in diversity between regions when spatial grain is increased (*88*). However, global diversity estimates made at fine resolutions may nonetheless have higher uncertainty, and thus, we examined how consistent our diversity patterns were over a range of increasing resolutions (*75*), similar to the methodology in “Comparison of richness estimates to community-level point observations.” For all taxa, we first coarsened each species’ range estimate raster (either polygonal range estimate or SDM prediction, based on data availability) to coarser resolutions of 20, 50, 100, 200, and 500–arc min resolutions (with bilinear resampling to the maximum value to ensure that maximum richness per cell did not change) using the Python package *rasterio (**89**)*. Therefore, if a window of four cells representing a cell of the coarser resolution contained suitability predictions of 0.2, 0.45, 0.9, and 0.1 (or binary values of 0, 0, 1, and 0), the resulting resampled raster would have a value of 0.9 (or 1 for binary values) for this cell. We chose this strategy so that if a suitable area exists within a larger region that contains a population, the larger region would also be categorized as suitable for the species because it contained some suitable area. We then made diversity estimates of the coarsened range estimates (using the same methodology as for the 10–arc min resolution data) to create a range of diversity estimates at coarser spatial grains. To assess how correlated each coarser ant diversity estimate was with the original 10–arc min estimate, we extracted the values for the centroids of the 10–arc min cells from the diversity estimates at all resolutions (10, 20, 50, 100, 200, and 500 arc min), removed any cells associated with NA values, and calculated Spearman’s rank correlation coefficients. We did the same comparison between diversity estimates for ants and vertebrates at progressively coarser resolutions and calculated correlation between richness values, as well as overlap proportion between diversity centers (figs. S4 and S5).

In summary, the diversity patterns we observed were quite robust to changes in spatial grain: Correlation between 10 arc min and coarser resolutions was above 0.9 for both ant richness and rarity except for 500 arc min, where a drop in correlation occurred to 0.89 for richness and 0.82 for rarity. We also assessed how correlated each vertebrate group’s diversity estimates were to the ant diversity estimate at the same resolution by calculating the Spearman’s rank correlation. We found that, in general, correlation increased with grain size for richness yet decreased slightly for rarity, and that overlap of diversity centers decreased slightly with grain size for richness yet remained mostly constant for rarity (fig. S3). For both richness and rarity at most resolutions, reptiles were most correlated with ants, while birds were least correlated (figs. S4 and S5).

### Predicting changes to diversity centers under a high-sampling scenario

We trained Random Forest models on our estimates of ant diversity to make predictions of undiscovered ant diversity under a scenario of equally high sampling across the globe. Specifically, we built separate models predicting our global estimates of ant richness and rarity (excluding Antarctica) as a function of climatic variables [19 bioclimatic variables from WorldClim 2.0 (*59*)], topography [Shuttle Radar Topography Mission (SRTM) elevation from WorldClim 2.0], categorical biogeographic realms (*81*), the GABI ant sampling density, and summed vertebrate richness or rarity, all at 10-arc min resolution. To avoid spurious variable importance values, we removed variables with high collinearity using the vifcor() function from the R package *usdm* (*83*) with a threshold of 0.7. We used slightly less restrictive criteria here than for the macroecological model (see “Alternative methods to estimate species richness”), as Random Forest is known to be relatively robust to multicollinearity (*90*), but this only resulted in the addition of one more bioclimatic variable [precipitation of the driest month (bio14)]. The biogeographical realms shapefile dataset we used from Holt *et al.* (*81*) lacked coverage of many smaller islands (particularly in Oceania) and some coastal areas, so we first converted the polygons to a raster and then performed a *k*-nearest neighbors classification with the R package *spatialEco* (*91*) to classify cells without data to the most proximal cell with a biogeographical classification. For our ant sampling density grid (see “Species range estimates”), low density reflects either lack of field collection and/or taxonomic work to process existing collections. The vertebrate diversity estimates were projected from the equal-area Eckert IV projected coordinate system to WGS 1984 (with bilinear resampling to the maximum value) to match the geographic coordinate system of the other datasets. We included vertebrate richness and rarity predictors for two reasons. First, it lets us determine the extent of predictive power that vertebrate biodiversity patterns hold beyond that captured by climate and region. Second, since these data represent the current state of knowledge of vertebrate biodiversity centers, they allow us to assess whether the ant data alone identify novel regions of richness and rarity even after including important regions for vertebrates.

We tuned model complexity and evaluated models using spatial cross-validation to optimize transferability to new conditions. Using the R package ranger (*92*), we built Random Forest models with different values of the hyperparameter “mtry,” which controls how many predictor variables are randomly sampled at each split within a tree. We used a range of 1 to 10—higher values indicate more variable interactions are allowed, leading to higher model complexity (*93*)—with other hyperparameters left at their defaults. After removing highly collinear predictor variables, we built models with the following variables: mean diurnal temperature range (bio2), mean temperature of wettest quarter (bio8), mean temperature of driest quarter (bio9), precipitation of driest month (bio14), precipitation seasonality (bio15), precipitation of warmest quarter (bio18), precipitation of coldest quarter (bio19), elevation, biogeographic realm, ant sampling density, and vertebrate richness or rarity. We log-transformed rarity values to avoid problems with model convergence due to extremely small values. To evaluate models, we implemented spatial cross-validation using a fivefold systematic 10 × 14 checkerboard partitioning scheme (fig. S6) using the R package blockCV (*94*) and then calculated the average mean square error (MSE) over the folds for each model. We performed model selection using spatial cross-validation as it tends to result in models with better transferability to new conditions (*38*). We selected optimal model complexity settings that resulted in the minimum average MSE (mtry = 4 for richness and mtry = 2 for rarity; fig. S7) and then trained models with these settings on the full dataset. For these models, we made maps to show the average root mean square error per block labeled by their spatial fold, which highlights areas that had high prediction error when the model was trained on the other spatial folds but missing the data from the fold represented by each block (fig. S7). We calculated variable importance values for each selected model with the permutation importance option in ranger (fig. S7). We then made extrapolations of richness and rarity for the globe under a scenario of equally high sampling by setting all cells with values in the sampling density grid to 1, representing the highest observed sampling density in our dataset (Figs. 3 to 6). Last, we overlaid the high-sampling scenario maps with the original diversity maps for ants and vertebrates and calculated percent overlap between them (Figs. 3 to 6).

### Global protected area coverage

To determine how much diversity center area is currently protected, we calculated the total area of global protected areas located within our diversity center estimations for each taxon (Fig. 7). We downloaded the World Database on Protected Areas 1.6 (*95*) file geodatabase from <www.protectedplanet.net> (downloaded 11 March 2021) and retained only designated and national protected area polygons from the feature dataset to match the methodology of Jenkins *et al.* (*27*). We then fully dissolved the multifeature polygon layer to a univalue, single feature without overlap and projected it to the equal-area coordinate system Eckert IV. We converted the diversity center rasters for all taxa and sampling extrapolations for ants into univalue polygons and then used them to mask the protected areas layer. All GIS operations were conducted with ArcGIS Pro 2.6.0 (*62*).

## Supplementary Materials

### This PDF file includes:

Figs. S1 to S7 Tables S1 to S4 Data S1

Correction (18 August 2022):
The descriptions for panels B and C in the caption of Figure S3 in the Supplementary Materials were erroneously swapped. The PDF and HTML have been updated. The edits do not affect the results or conclusions of the manuscript. The original version is available here:
