# Supplementary material for: The global distribution of known and undiscovered ant biodiversity
Source: Sci Adv. 2022 Aug 3;8(31):eabp9908. doi: 10.1126/sciadv.abp9908 (PMC9348798; doi:10.1126/sciadv.abp9908)
Supplement: Supplementary file 2 — Correction (18 August 2022): The descriptions for panels B and C in the caption of Figure S3 in the Supplementary Materials were erroneously swapped. The PDF and HTML have been updated. The edits do not affect the results or conclusions of the manuscript. The original version is available here: [file sciadv.abp9908_sm.v1.pdf]

Science Advances
AAAS

Supplementary Materials for

The global distribution of known and undiscovered ant biodiversity

Jamie M. Kass *et al.*

Corresponding author: Evan P. Economo, evaneconomo@gmail.com; Jamie M. Kass, jamie.m.kass@gmail.com

Sci. Adv. **8**, eabp9908 (2022)
DOI: 10.1126/sciadv.abp9908

The PDF file includes:

Figs. S1 to S7
Tables S1 to S4
Legend for data S1

Other Supplementary Material for this manuscript includes the following:

Data S1

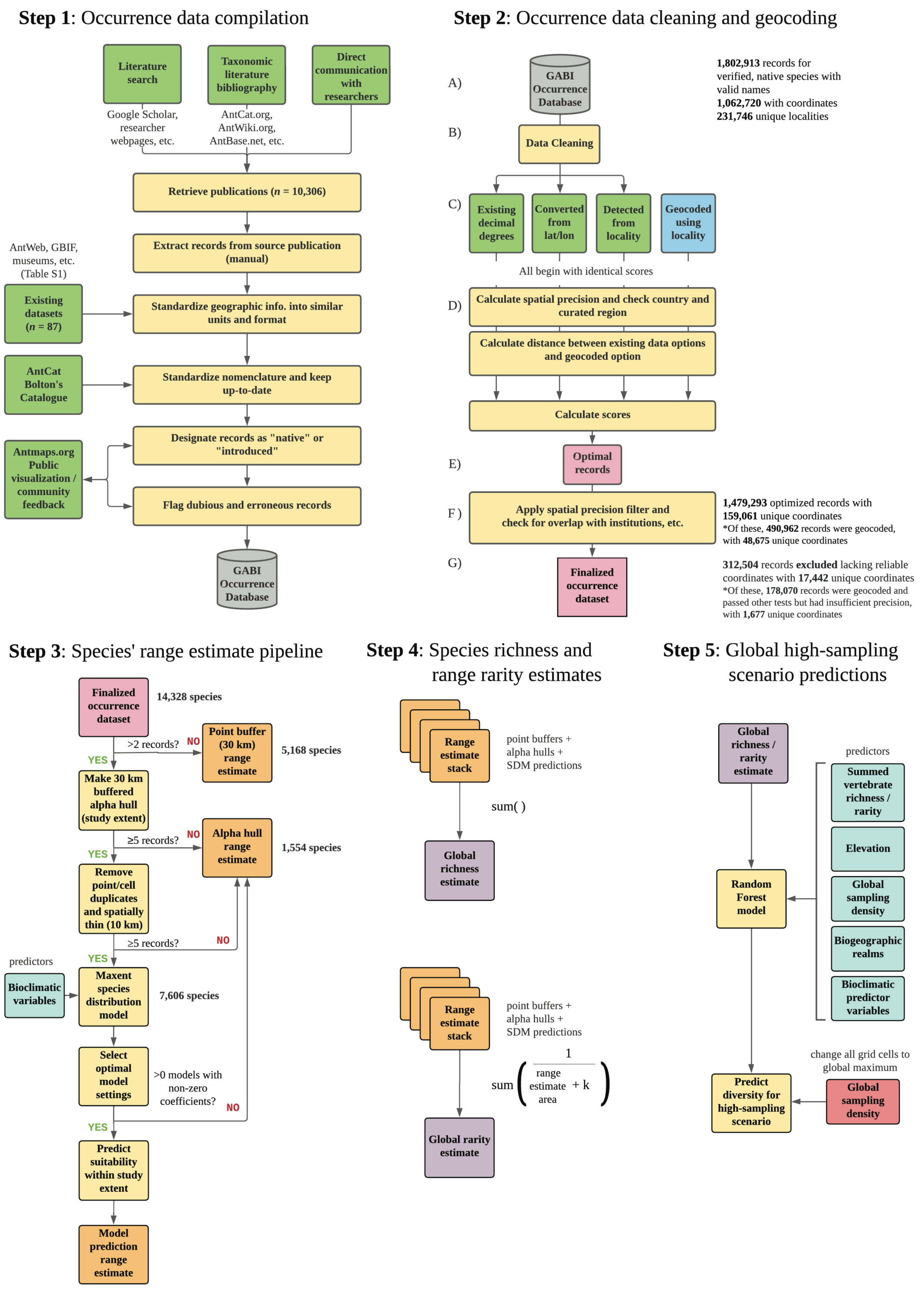

Fig. S1. Overview of the analysis workflow. The analyses in this paper involve data compilation, data cleaning and geocoding, estimating ranges, estimating richness and rarity, and predicting under a high-sampling scenario. The first step (data compilation) has been an ongoing effort over many years (see ref. 21) rather than a procedure applied once for this paper, while the others are specifically designed for this study.

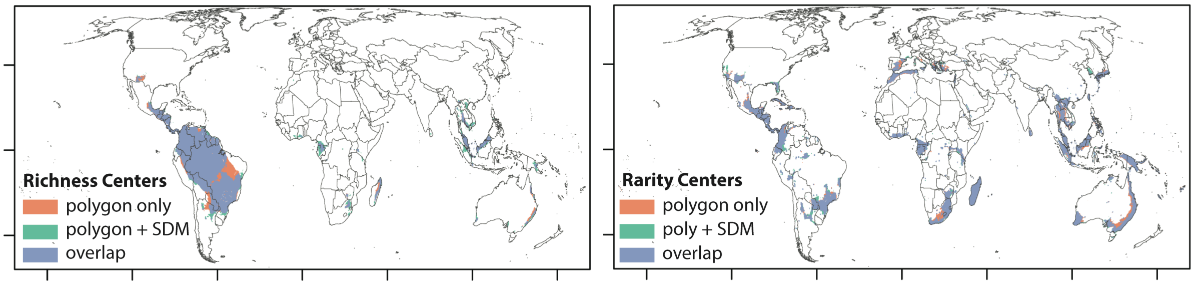

Fig. S2. Comparing richness and rarity centers inferred with environmental-based SDMs of species ranges versus polygons alone. Our primary analysis method used buffered points or alpha hull polygons to represent range estimates for all species, but then estimated suitability within these polygons for species with 5 records or greater using species distribution models (SDMs) fit with climatic predictor variables (on the map "polygon + SDM"). This modeling step helped us make more conservative range estimates that assigned areas within their range extent (alpha hull) lower weight outside species' modeled environmental affinities. However, we determined the extent to which using these models influences the final richness and rarity centers by mapping overlap between the "polygon + SDM" results with richness/rarity centers inferred by stacking univalue polygons assuming the species is found everywhere within its alpha hull ("polygon only" on map), and found mostly marginal effects on the final result.

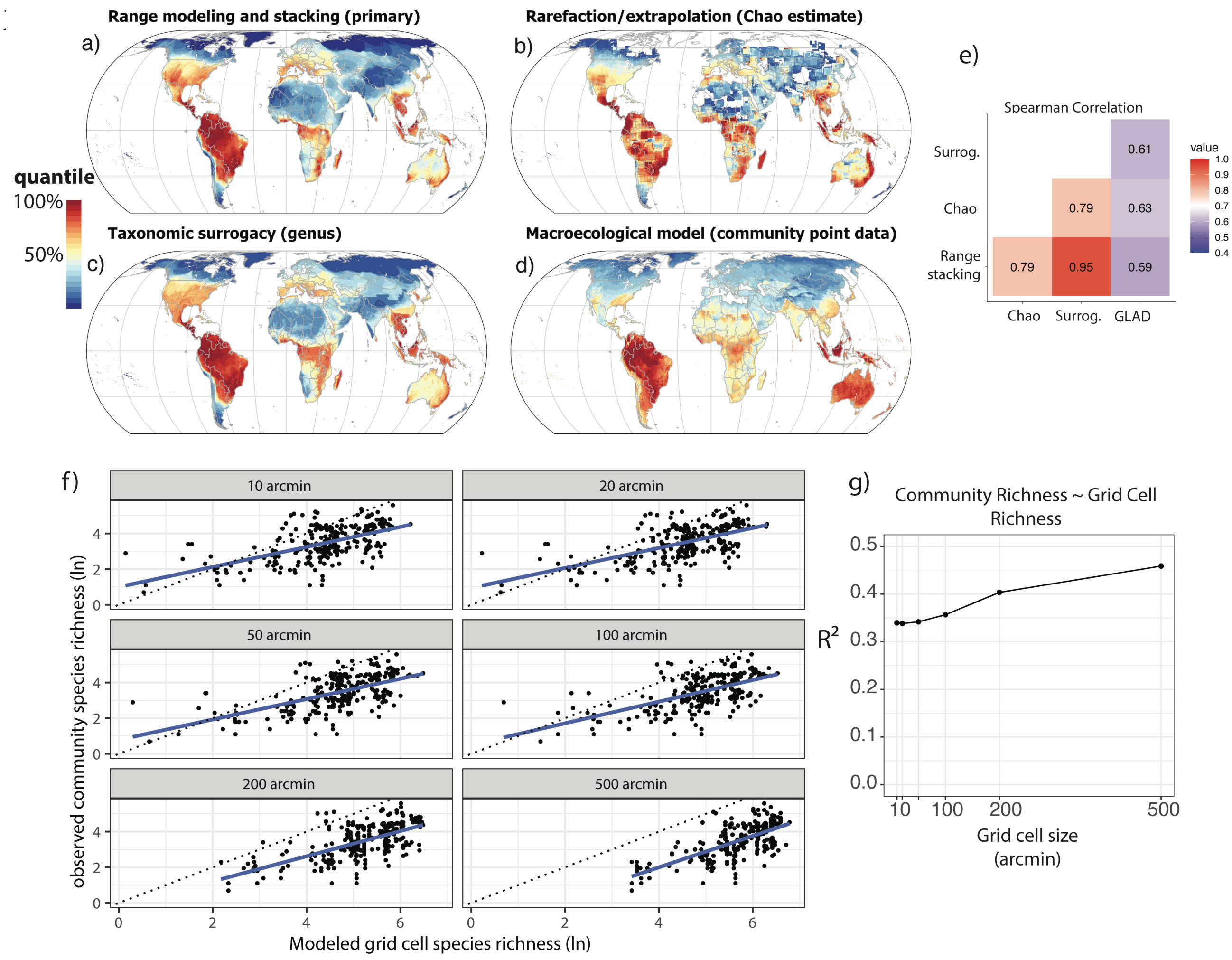

Fig. S3. Richness estimation methods comparison. a) Our primary methodology for estimating richness was to estimate the range of each species individually, then stack them together. However, to assess sensitivity to methodological choices, we also estimated richness with three other methods, including b) taxonomic surrogacy by modeling the ranges of genera and predicting richness from the empirical species-genus richness correlation, c) using a rarefaction/extrapolation approach on occurrence data in a moving window (which does not require range modeling), and d) a macroecological model that estimates relationships between macroscale predictor variables and point community richness estimates from the Global Ants Database (GLAD). e) All methods are highly correlated, although the point richness model is less correlated than the others. f) We also compared our grid cell predictions with maximum GLAD point richness measured in each cell. The latter, unlike our global dataset of described species, reflects richness including morphospecies. In general, maximum point observations were lower than the predicted richness for the whole grid cell, which was expected given that a community will rarely contain all species in a region. Likewise, even though the point estimates contain morphospecies, they are rarely higher than modeled values, supporting the notion that our estimates are not exceedingly low even though they do not include undescribed taxa. g) R-squared from the correlation is not strongly influenced by choice of resolution of gridding process.

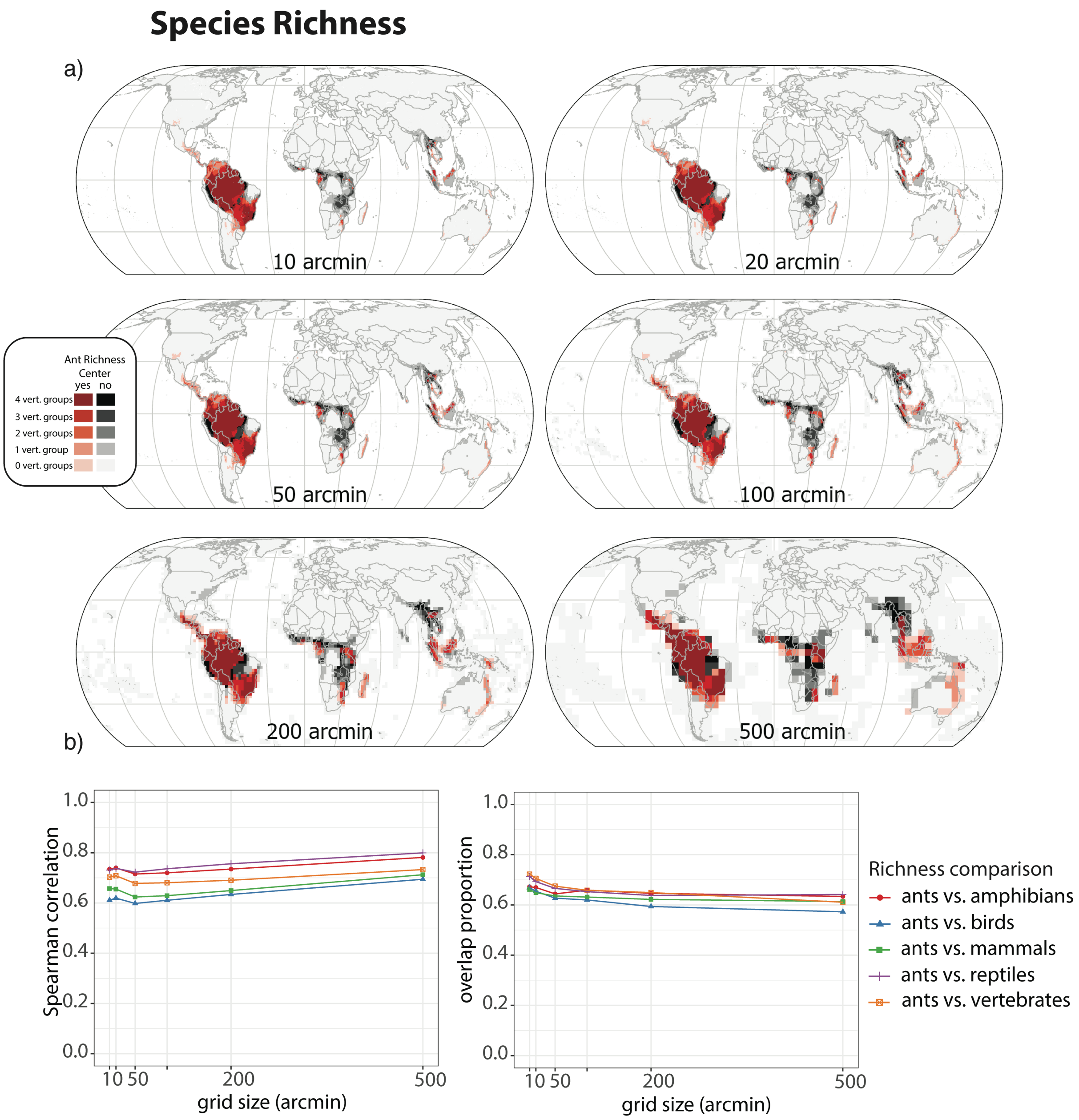

Fig. S4. Robustness of ant richness centers to analysis scale. We examined the influence of grid cell size on diversity center distribution and congruence. a) In general, richness center locations were stable until the largest 500 km grid size, at which point centers began to disappear, although the detailed structure of each center naturally depends to some extent on spatial resolution. b) Spearman correlation of richness values and fraction overlap of top 10% areas between ants and other taxa are largely insensitive to the scale of the analysis.

Rarity

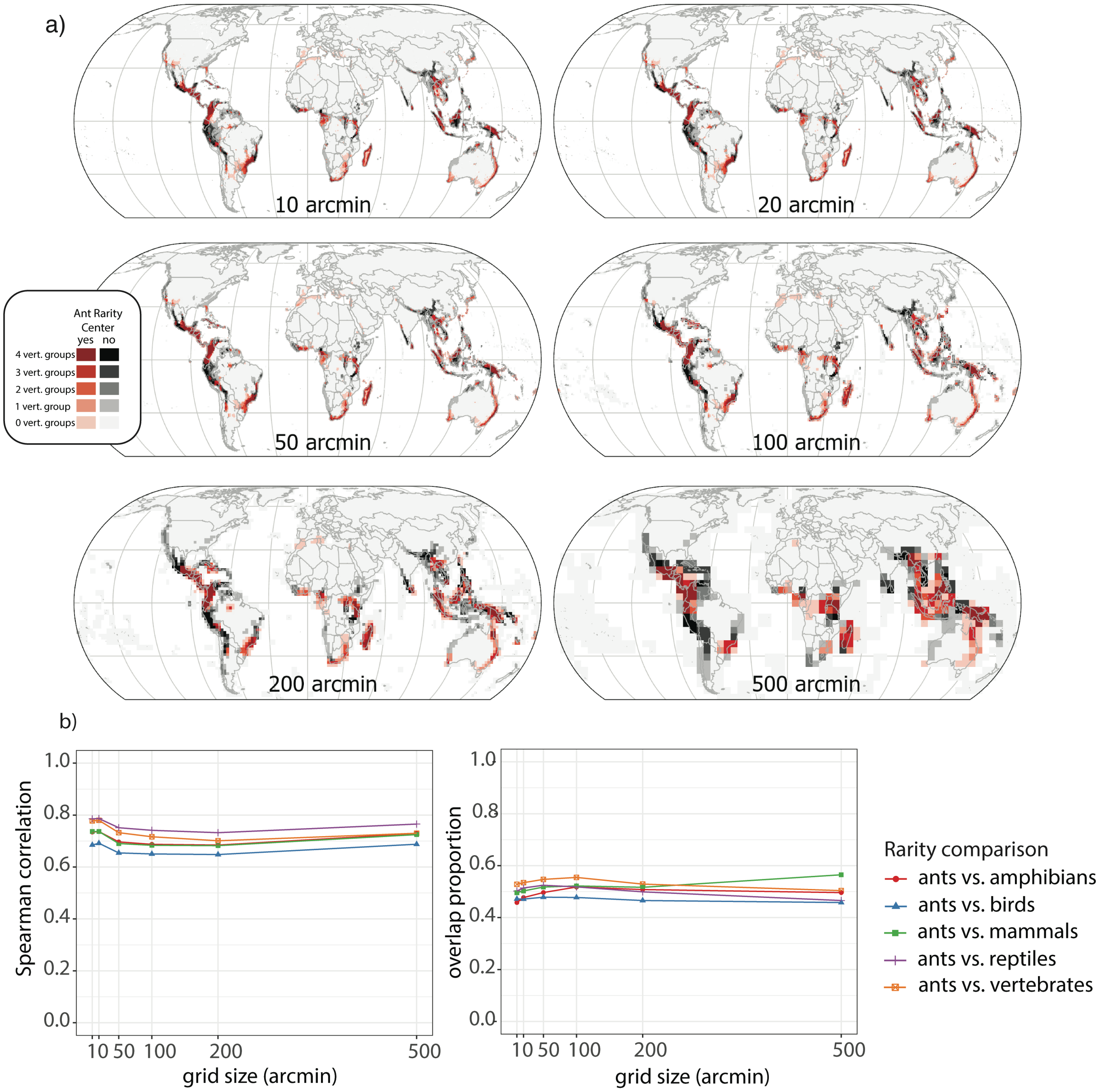

Fig. S5. Robustness of ant rarity centers to analysis scale. We examined the influence of grid cell size on rarity center distribution and congruence. a) In general, rarity center locations were stable until the largest 500 km grid size, at which point rarity centers began to disappear, although the detailed structure of each center naturally depends to some extent on spatial resolution. b) Spearman correlation of richness values and fraction overlap of top 10% areas between ants and other taxa are largely insensitive to the scale of the analysis.

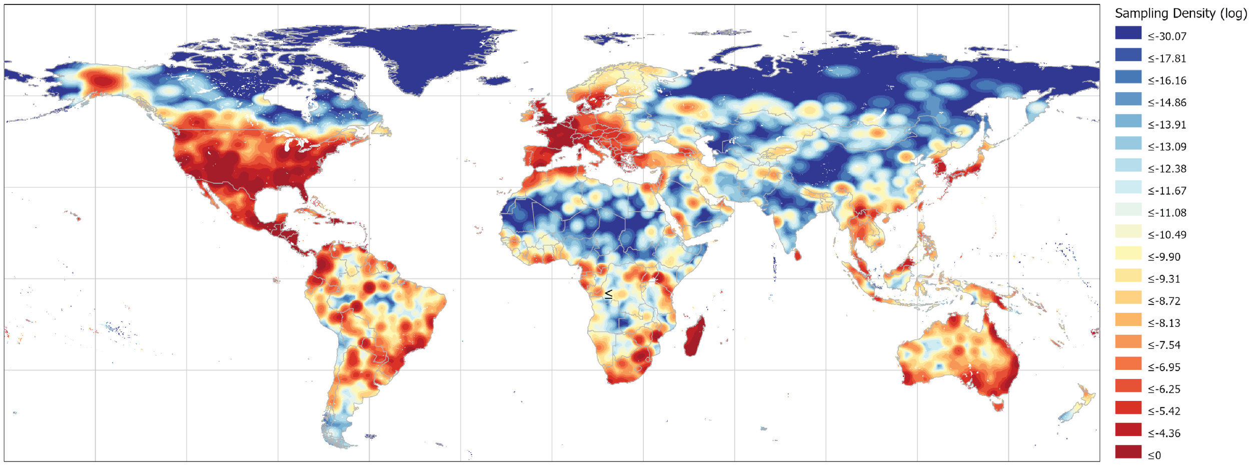

Fig. S6. Global variation in ant occurrence record density. Occurrence data density (# of records for described species) was smoothed to create a grid of sampling bias used both to account for bias in the individual species distribution models and as a predictor variable for the Random Forest diversity models.

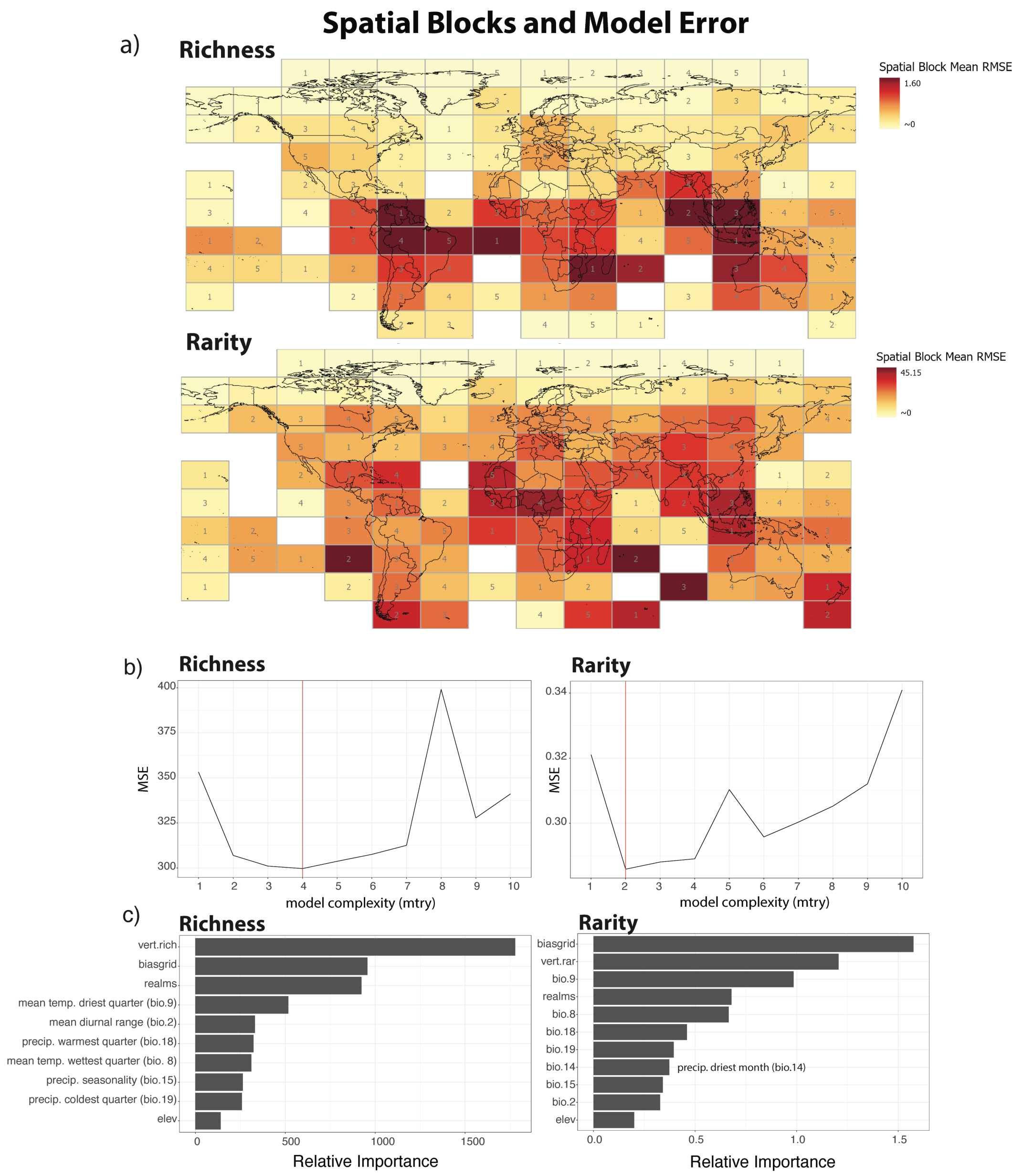

Fig. S7. Random Forest model cross-validation, tuning, and variable importance. To evaluate the Random Forest models, we used a spatial cross-validation procedure that separates the globe into systematically defined spatial blocks that delineate training and validation datasets. This evaluation procedure helped us select optimal complexity settings for our models. a) These maps show the spatial fold (k = 5) assigned to each block we used for cross-validation and the root mean square error (RMSE) associated with the selected model for each individual block. For example, high RMSE for a block in fold 1 means that the selected model trained on folds 2–5 had high error when predicting richness for the conditions in that block. It is important to state that although the complexity settings for models were chosen via cross-validation, final models were trained on the full dataset—thus, these RMSE values are not reflective of the final models' performance. b) We fitted models with a range of complexity settings and selected optimal settings with maximum predictive ability (minimum mean square error) for richness and rarity. c) The permuted variable importance scores for the optimized models. These models were then used to predict changes in richness and rarity with universal high sampling around the globe.

Table S1.
The number of records and percent of total records originating from different data sources that were compiled for the Global Ant Biodiversity Informatics (GABI) database (downloaded April 17, 2021). Relevant abbreviations for institutions are in parentheses, aggregator database sources are in brackets, and personal datasets are also noted in parentheses with date of access. All data used are available in the supplemental archive (https://doi.org/10.5061/dryad.wstqjq2pp).

Data Source	Number records	% Total Records
Literature (10,306 Publications)	897509	35.86
AntWeb	585075	23.38
Museum of Comparative Zoology (MCZ), Harvard University (entomological collection)	124694	4.98
iDigBio	120595	4.82
Mississippi Entomological Museum (MEM)	83862	3.35
INBio Collection [GBIF]	50644	2.02
Antarea	48592	1.94
CSIRO Collection	45125	1.80
Bees, Wasps and Ants Recording Society	43294	1.73
Field Museum of Natural History (FMNHiNS)	33760	1.35
William Mackay Collection (personal dataset; accessed 2016).	30645	1.22
Formidabel Database	27234	1.09
Museum of Comparative Zoology, Harvard University [GBIF]	26152	1.04
J. T. Longino Collection Database (personal dataset; accessed 2010).	22501	0.899
IZIKO South Africa Museum Collection	21621	0.864
Dattilo W. et al. 2019. <https://doi.org/10.1002/ecy.2944>	20210	0.807
Centre for Biodiversity Genomics (BiOUG)	19985	0.798
Texas A&M University Insect Collection (ENTO)	15661	0.626
Australian Museum provider for Online Zoological Collections of Australian Museums (OZCAM) [GBIF]	13132	0.525
AFRC (AfriBugs Collection, Pretoria)	12323	0.492
Zoological Museum, Natural History Museum of Denmark [GBIF]	12147	0.485
Instituto de Ciencias Naturales de la Universidad Nacional de Colombia [GBIF]	12086	0.483
Triplehorn Insect Collection (OSUC), Ohio State University [GBIF]	11570	0.462
iNaturalist (research-grade observations)	11495	0.459
Instituto Nacional de Pesquisas de Amazonia [GBIF]	11252	0.450
Texas Tech University – Invertebrate Zoology (TTU-Z)	11111	0.444
Field Museum of Natural History [GBIF]	10069	0.402
Archbold Biological Station Arthropod Collection (ARTHARCH)	9948	0.397
Royal Belgian Institute of Natural Sciences (from Paraguayan dry Chaco) [GBIF]	8846	0.353
Brigham Young University Arthropod Museum (BYUC)	8230	0.329
C.A. Triplehorn Insect Collection, Ohio State University	8116	0.324
University of Arizona Insect Collection (UAiC)	7740	0.309

Robson Simon Ant Collection (private dataset; accessed 2014)	7499	0.300
Illinois Natural History Survey [GBIF]	7402	0.296
Symbiota Collections of Arthropods Network Project (SCAN)	6765	0.270
University of Colorado Museum of Natural History Entomology Collection (UCMC)	6642	0.265
Johnson, R. (personal dataset; accessed 2014) <http://www.asu.edu/clas/sirgtools/resources.htm>	5877	0.235
BugGuide	5322	0.213
C.A. Triplehorn Insect Collection, Ohio State University (OSUC)	5209	0.210
Colorado Plateau Museum of Arthropod Biodiversity (CPMAB)	5033	0.201
ArtDatabanken Bugs (via GBIG)	4603	0.184
Des Lauriers, J. (personal dataset; accessed 2020)	4584	0.183
Lubertazzi, D. Museum of Comparative Zoology (MCZ) at Harvard University (personal dataset)	4430	0.177
University of Hawaii Insect Museum (UHiM)	4297	0.172
Zoologisches Forschungsinstitut und Museum Alexander Koenig [GBIF]	4127	0.165
New Mexico State Collection of Arthropods (NMSU)	3997	0.160
Museo de Entomología de la Universidad del Valle [GBIF]	3929	0.157
UAM Entomology Collection (Arctos) [GBIF]	3760	0.150
Arizona State University Hasbrouck Insect Collection (ASUHiC)	3481	0.139
The Sam Noble Museum Department of Recent Invertebrates (RiNVRT)	3182	0.127
Smithsonian Institution, National Museum of Natural History (entomological collection)	3174	0.127
Legakis A. Collection Database, provided by Georgiadis C. (private dataset; accessed 2015)	2365	0.094
BioFokus [Artsdatabanken]	2271	0.091
The University of Central Florida Collection of Arthropods (UCFC)	2208	0.088
Guenard, B. & Liu C., Xishuangbanna Tropical Botanical Garden, Yunnan, China (personal dataset, accessed 2013)	2110	0.084
University of Guam Insect Collection (ESUG)	1868	0.075
Norsk Institutt for Naturforskning [Artsdatabanken]	1680	0.067
Cleveland Museum of Natural History (CMNHENT) [GBIF]	1580	0.063
Tinault A. Database [GBIF]	1416	0.057
Norsk Entomologisk Forening [Artsdatabanken]	1353	0.054
Museum of Southwestern Biology, Division of Arthropods (MSBA)	1212	0.048
Canadensys Database	1185	0.047
Donoso D. (personal dataset; accessed 2014)	1153	0.046
Insect Biodiversity and Biogeography Laboratory, Hong Kong	1033	0.041
Mirmecofauna de la reserva ecologica de San Felipe Bacalar [GBIF]	818	0.033
Essig Museum of Entomology (EMEC)	716	0.029
MUST [Artsdatabanken]	713	0.028
Koch Sheard J. 2020. <https://doi.org/10.15468/dcijnc> [GBIF]	674	0.027
Essig Museum of Entomology – SCAN (PKPC)	456	0.018
Escuela Politecnica Nacional, Ecuador	440	0.018

United States Geological Survey, Patuxent Wildlife Research Center, Native Bee Inventory and Monitoring Lab	439	0.018
Prince Edward Island Museum and Heritage Foundation	364	0.015
Naturhistorisk Museum – UiO [Artsdatabanken]	344	0.014
University of Kansas Natural History Museum Entomology Division (SEMC)	333	0.013
Menke, S.B. Field Museum of Natural History specimen data from	320	0.013
Catálogo de insectos de la colección del Centro de Entomología – SCAN (CEAM)	314	0.013
The Albert J. Cook Arthropod Research Collection, Michigan State University. http://www.arc.ent.msu.edu:8080/collection/index.jsp (accessed 2014)	289	0.012
Colorado Plateau Museum of Arthropod Biodiversity (PiSP)	254	0.010
University of Alberta Museums, E. H. Strickland Entomological Museum (UASM)	232	0.009
University of California Santa Barbara Invertebrate Zoology Collection (iZC)	222	0.009
The Albert J. Cook Arthropod Research Collection (MSUC)	220	0.009
Colorado Plateau Museum of Arthropod Biodiversity (CACH)	204	0.008
University of Delaware Insect Research Collection (UDCC)	204	0.008
Dugway Proving Ground Natural History Collection (DUG-ENT)	134	0.005
Tromso Museum – Universitetsmuseet [Artsdatabanken]	109	0.0044
Colorado Plateau Museum of Arthropod Biodiversity (MEVE)	106	0.0042
Ohio State Acarology Laboratory, Ohio State University (OSAL)	102	0.0041
Colorado Plateau Museum of Arthropod Biodiversity (CANY)	99	0.0040
The Davidson College Entomology Collection (DCEC)	80	0.0032
NAU Forest Entomology Collection (NAUF5F)	80	0.0032
Colorado Plateau Museum of Arthropod Biodiversity (GEWA)	65	0.0026
C.P. Gillette Museum of Arthropod Diversity (CSUC)	49	0.0020
NTNU Vitenskapsmuseet [Artsdatabanken]	44	0.0018
GBIF noder utenfor Norge [Artsdatabanken]	43	0.0017
Colorado Plateau Museum of Arthropod Biodiversity (GCRA)	43	0.0017
Colorado Plateau Museum of Arthropod Biodiversity (ZiON)	41	0.0016
Museum of Northern Arizona – Grand Canyon National Park Collection (GRCA)	36	0.0014
University of Puerto Rico Mayagüez Invertebrate Collection (iNVCOL)	32	0.0013
Museum of Northern Arizona - Walnut Canyon National Monument Collection (WACA)	17	0.0007
The Broward College Insect Collection (BCiC)	16	0.0006
The University of Texas at El Paso Biodiversity Collections, entomology collection (CZUG)	6	0.00024
Booher, D. (personal dataset; accessed 2014)	4	0.00016
Universitetsmuseet i Bergen (UiB) [Artsdatabanken]	2	0.00008
Sarnat, E. (personal dataset; accessed 2015)	2	0.00008
Denver Botanic Gardens Collection of Arthropods (DBGA)	1	0.00004
Hoffmann, B. USDA Honolulu Collection. (personal dataset; accessed 2020)	1	0.00004

Table S2.
Spearman and Pearson correlation values for richness and rarity based on raster overlays of global richness estimates. Here, "RF" refers to Random Forest model extrapolations under a global high-sampling scenario, and "no vert" refers to the absence of a predictor variable in the RF model for vertebrate richness/rarity. Due to the large number of data points considered, confidence intervals for correlation values were extremely narrow (on the order of ±0.001) and are not reported here.

Taxon 1	Taxon 2	Spearman richness	Pearson richness	Spearman rarity	Pearson rarity
ants	amphibians	0.735	0.752	0.734	0.596
ants	reptiles	0.73	0.738	0.785	0.634
ants	birds	0.611	0.688	0.684	0.503
ants	mammals	0.657	0.732	0.736	0.596
ants	vertebrates	0.703	0.75	0.777	0.636
ants	ants RF	0.722	0.72	0.912	0.831
ants	ants RF no vert	0.736	0.687	0.87	0.746
amphibians	reptiles	0.742	0.821	0.758	0.622
amphibians	birds	0.839	0.836	0.839	0.66
amphibians	mammals	0.848	0.842	0.839	0.7
amphibians	ants RF	0.695	0.685	0.784	0.708
amphibians	ants RF no vert	0.663	0.592	0.737	0.62
reptiles	birds	0.667	0.796	0.777	0.677
reptiles	mammals	0.668	0.805	0.81	0.702
reptiles	ants RF	0.877	0.819	0.879	0.771
reptiles	ants RF no vert	0.822	0.728	0.855	0.685
birds	mammals	0.893	0.922	0.878	0.822
birds	ants RF	0.638	0.717	0.79	0.759
birds	ants RF no vert	0.549	0.592	0.745	0.695
mammals	ants RF	0.616	0.741	0.819	0.809
mammals	ants RF no vert	0.549	0.633	0.768	0.749
vertebrates	ants RF	0.726	0.778	0.877	0.857
vertebrates	ants RF no vert	0.649	0.66	0.829	0.775
ants RF	ants RF no vert	0.96	0.95	0.978	0.954

Table S3.
Quantile values used to define diversity centers (90%) and upper levels of diversity for continuous maps (99%).

Estimate	Taxon	90%	99%
Species richness	Ant	111.139	230.549
	Amphibian	37	93
	Reptile	107	167
	Bird	338	504
	Mammal	125	179
	Vertebrate	601	917
	Chao estimator	560.707	947.654
	Genus surrogate	134.724	254.562
	Macroecological model	75.143	140.879
	Macroecological model, clamped	74.894	140.339
	Random Forest extrapolation	241.808	315.602
	Random Forest extrapolation, no vertebrate richness predictor	227.518	332.305
Range rarity richness	Ant	1.14×10^{-4}	4.46×10^{-4}
	Amphibian	3.67×10^{-5}	1.50×10^{-4}
	Reptile	8.10×10^{-5}	2.25×10^{-4}
	Bird	1.36×10^{-4}	4.18×10^{-4}

	Mammal	5.99×10^{-5}	1.56×10^{-4}
	Vertebrate	3.09×10^{-4}	8.81×10^{-4}
	Random Forest extrapolation	2.28×10^{-4}	6.04×10^{-4}
	Random Forest extrapolation, no vertebrate richness predictor	3.17×10^{-4}	7.42×10^{-4}

Table S4.

Overlap values for richness and rarity based on rasters of diversity centers for global richness estimates. Here, "RF" refers to Random Forest model extrapolations under a global high-sampling scenario, and "no vert" refers to the absence of a predictor variable in the RF model for vertebrate richness/rarity.

Taxon 1	Taxon 2	Overlap richness	Overlap rarity
ants	amphibians	0.672	0.458
ants	reptiles	0.713	0.502
ants	birds	0.669	0.472
ants	mammals	0.661	0.496
ants	vertebrates	0.722	0.528
ants	ants RF	0.538	0.661
ants	ants RF no vert	0.434	0.557
amphibians	reptiles	0.72	0.467
amphibians	birds	0.744	0.598
amphibians	mammals	0.689	0.609
amphibians	ants RF	0.432	0.6
amphibians	ants RF no vert	0.352	0.5
reptiles	birds	0.688	0.565
reptiles	mammals	0.671	0.564
reptiles	ants RF	0.542	0.596
reptiles	ants RF no vert	0.434	0.476
birds	mammals	0.742	0.742
birds	ants RF	0.438	0.726
birds	ants RF no vert	0.345	0.589
mammals	ants RF	0.487	0.684
mammals	ants RF no vert	0.406	0.586
vertebrates	ants RF	0.465	0.786
vertebrates	ants RF no vert	0.384	0.62
ants RF	ants RF no vert	0.736	0.763

Data S1. (separate files)

The supplemental data package containing all data, analysis code, and results is available in a Dryad archive (https://doi.org/10.5061/dryad.wstqjq2pp).
